# Characterization of *Pseudomonas alliivorans* strains isolated from Georgia, USA: insights into genomic diversity and pathogenicity in onions

**DOI:** 10.1128/aem.01643-25

**Published:** 2025-11-25

**Authors:** Mei Zhao, Michelle Pena Maclellan, Anuj Lamichhane, Sujan Paudel, Ron Gitaitis, Brian Kvitko, Bhabesh Dutta

**Affiliations:** 1 Department of Plant Pathology, College of Plant Protection, China Agricultural University 34752, Beijing, People's Republic of China; 2 Department of Plant Pathology, University of Georgia 117299, Tifton, Georgia, USA; 3 Department of Plant Pathology, University of Georgia 1355, Athens, Georgia, USA; The University of Tennessee Knoxville, Knoxville, Tennessee, USA

**Keywords:** Hrp1 type III secretion system, *Pseudomonas syringae*, rhizobium type III secretion system, phylogenetic analysis, horizontal gene transfer

## Abstract

*Pseudomonas alliivorans* is an important emerging pathogen affecting numerous crops. The species is closely related to *Pseudomonas viridiflava,* with which *P. alliivorans* strains were often misidentified in the past. Here, we investigated the genetic and pathogenic characteristics of *P. alliivorans* strains isolated primarily from onions and weeds in Georgia, USA, using whole-genome sequencing, comparative genomics, and functional assays. We delineated the core genome and genetic diversity of these isolates, assessed their pathogenicity on onion foliage and red onion scales, and examined the roles of key virulence determinants (Hrp1-type III secretion system [T3SS], rhizobium-T3SS, type II secretion systems [T2SSs], and thiosulfinate [allicin]-tolerance *alt* cluster). Our results showed that the Hrp1-T3SS is pivotal for pathogenicity in *P. alliivorans*, whereas the rhizobium-T3SS, T2SSs, and *alt* cluster do not contribute to symptom development on red onion scales. Notably, the *alt* cluster confers *in vitro* thiosulfinate tolerance, supporting bacterial survival against onion-derived antimicrobial compounds. Additionally, homologous recombination in *P. alliivorans* occurs infrequently (at approximately one-tenth the rate of point mutations) and involves divergent DNA segments. The *alt* cluster is acquired through horizontal gene transfer, as evidenced by its lower GC content and the presence of adjacent transposases. In summary, our research provides valuable insights into the genetic diversity, evolutionary dynamics, and virulence mechanisms of *P. alliivorans* strains from Georgia, USA.

**IMPORTANCE:**

*Pseudomonas alliivorans* is an emerging plant pathogen that threatens onion and other plants of economic importance. This study identifies key traits that help this bacterium cause disease, such as a specific secretion system critical for infecting onions, and a gene cluster that aids bacterial survival in onion tissues. Beyond highlighting weed as a potential inoculum source and supporting better weed management, the findings of this research open avenues for more targeted disease menegement. By unraveling the genetics of this pathogen, we can develop improved ways to detect, prevent, and reduce its impact, protecting crop health and yields.

## INTRODUCTION

Onions (*Allium cepa* L.) are one of the economically most important vegetable crops globally, providing important nutrients and enhancing culinary diversity (1). Despite their economic significance, onion cultivation is confronted with formidable challenges from various bacterial pathogens, which can have devastating effects on crop yield, quality, and marketability. Some of the bacterial pathogens that onion growers must contend with include *Pantoea* spp., *Xanthomonas euvesicatoria* pv. *allii, Burkholderia* spp., *Pseudomonas* spp., and *Rouxiella badensis* (2–6). Among these pathogens, *Pseudomonas alliivorans* has recently emerged as a concern for onion growers; however, its characteristics remain relatively understudied (7). The dissemination and detrimental effects of these bacterial pathogens emphasize the pressing need for effective management strategies. Accurate identification and a deeper understanding of these bacterial pathogens are critical for effective disease management.

Managing these pathogens presents a complex set of challenges, including the absence of genetic resistance in commercial cultivars, the emergence of novel pathogens, tolerance to conventional bactericides (copper products), and the lack of effective and sustainable management options. The intricate interplay among bacterial pathogens, onions, and environmental factors further complicates efforts to manage these diseases. Given the substantial economic impact of bacterial pathogens on onion production, it is essential to enhance our knowledge of their epidemiology, interactions with hosts, and genetic diversity.

The genus *Pseudomonas*, comprising over 250 species, exhibits extraordinary ecological diversity, with roles ranging from plant-beneficial activities (e.g., growth promotion and biocontrol) to opportunistic or specialized plant pathogenic lifestyles (List of Prokaryotic Names with Standing in Nomenclature; https://lpsn.dsmz.de/genus/pseudomonas). Among the pathogenic members, certain species and strains pose a substantial threat to global food production and security, making it critical to understand the genetic diversity and pathogenic potential of these lineages. The resurgence or emergence of pathogenic *Pseudomonas* species, including *P. alliivorans*, highlights this necessity. Importantly, our prior taxonomic work (7) confirmed using 16S rRNA gene sequencing, multi-locus sequence analysis of 86 housekeeping genes, and core-proteome phylogeny that *P. alliivorans* clusters within the *Pseudomonas syringae* species complex, with *Pseudomonas viridiflava* (a well-documented member of this complex) as its closest known relative (7). Within the diverse genus *Pseudomonas*, strains of the *P. syringae* species complex exhibit considerable variability, with 60 documented pathovars (8–13). Studies have classified the *P. syringae* complex into at least nine genomospecies using DNA-DNA hybridization and 13 phylogroups using multi-locus sequence typing (14–17). These phylogroups are further divided into primary phylogroups (1, 2, 3, 4, 5, 6, and 10), which comprise major agricultural strains, and secondary phylogroups (7, 8, 9, 11, 12, and 13), which include more divergent, often understudied strains, such as *P. alliivorans* (14–17).

Despite its recent recognition as a distinct species, *P. alliivorans* remains insufficiently characterized with respect to its genetic diversity and pathogenic potential in onion (7). This study aims to address this knowledge gap by conducting a comprehensive characterization of *P. alliivorans* strains isolated from Georgia, USA. The goal is to understand the genetic basis of their ecological adaptation and to identify specific genes that may play roles in plant-microbe interactions. Specifically, our research aims to identify the virulence determinants that contribute to onion pathogenicity in *P. alliivorans*. By doing so, we hope to facilitate the development of targeted approaches for pathogen diagnostics and disease management in onions.

## MATERIALS AND METHODS

### Sample collection and bacterial isolation

We selected 110 *P*. *alliivorans* strains isolated from onion foliage and plant tissues of other hosts in Georgia, USA, for whole genome sequencing. The strain name, geographic location of isolation, year of isolation, and host of isolation are presented in Table 1. For isolation from symptomatic plants, infected tissues were disinfected with 70% ethanol and rinsed once with sterile distilled water. For weed samples without obvious symptoms, plant tissues were soaked in 10 mM phosphate-buffered saline while shaking overnight. A loopful of the resulting suspension was streaked onto nutrient agar (NA) plates for isolation and incubated at 28°C for 2 days. Dominant colonies were sub-cultured on NA plates and incubated for an additional 2 days for purification. Purified colonies were grown in liquid nutrient broth with shaking for 48 h, then stored in 15% (wt/vol) glycerol at −80°C for long-term storage.

**TABLE 1:** Origin and general features of *P. alliivorans* strains used in this study^*a*^

Strain	Location	Year	Host	Number of *alt* clusters	Pathogenicity on onion	Hypersensitivity reaction
20GA0068	Tattnall Co., GA	2020	*Allium cepa*	1	Positive	Positive
20GA0069	Tattnall Co., GA	2020	*Allium cepa*	1	Positive	NT
20GA0070	Tattnall Co., GA	2020	*Allium cepa*	1	Positive	NT
20GA0080	Toombs Co., GA	2020	*Allium cepa*	1	Positive	NT
20GA0081	Toombs Co., GA	2020	*Allium cepa*	0	Positive	NT
20GA0082	Toombs Co., GA	2020	*Allium cepa*	0	Positive	NT
20GA0083	Toombs Co., GA	2020	*Allium cepa*	0	Positive	NT
20GA0084	Tattnall Co., GA	2020	*Carolina geranium*	0	Positive	NT
20GA0148	Tattnall Co., GA	2020	*Allium cepa*	1	Positive	NT
20GA0149	Tattnall Co., GA	2020	*Allium cepa*	1	Positive	NT
20GA0198	Toombs Co., GA	2020	*Allium cepa*	0	Positive	NT
20GA0201	Toombs Co., GA	2020	*Allium cepa*	1	Positive	NT
20GA0207	Toombs Co., GA	2020	*Allium cepa*	0	Positive	NT
20GA0227	Tattnall Co., GA	2020	*Carolina geranium*	0	Positive	NT
20GA0228	Tattnall Co., GA	2020	*Carolina geranium*	0	Positive	NT
20GA0233	Tattnall Co., GA	2020	*Carolina geranium*	0	Positive	NT
20GA0235	Toombs Co., GA	2020	*Carolina geranium*	0	Positive	NT
20GA0237	Tattnall Co., GA	2020	*Carolina geranium*	1	Positive	NT
21GA0411	Toombs Co., GA	2021	Weed leaf	1	Positive	NT
21GA0412	Toombs Co., GA	2021	*Allium cepa*	1	Positive	NT
21GA0416	Toombs Co., GA	2021	*Allium cepa*	1	Positive	NT
21GA0420	Tattnall Co., GA	2021	Weed leaf	1	Positive	NT
21GA0421	Tattnall Co., GA	2021	Weed leaf	0	Positive	NT
21GA0425	Tattnall Co., GA	2021	Weed leaf	0	Positive	NT
21GA0426	Tattnall Co., GA	2021	Weed leaf	0	Positive	NT
21GA0427	Tattnall Co., GA	2021	Weed leaf	0	Positive	NT
21GA0475	Montgomery Co., GA	2021	Weed leaf	0	Positive	NT
21GA0476	Montgomery Co., GA	2021	Weed leaf	0	Positive	NT
21GA0477	Tattnall Co., GA	2021	Weed leaf	0	Positive	NT
21GA0478	Tattnall Co., GA	2021	Weed leaf	0	Positive	NT
21GA0479	Tattnall Co., GA	2021	Weed leaf	0	Positive	NT
21GA0480	Tattnall Co., GA	2021	*Allium cepa*	1	Positive	NT
21GA0481	Tattnall Co., GA	2021	*Allium cepa*	1	Positive	NT
21GA0482	Tattnall Co., GA	2021	*Allium cepa*	1	Positive	NT
21GA0483	Tattnall Co., GA	2021	*Allium cepa*	1	Positive	NT
21GA0484	Tattnall Co., GA	2021	*Allium cepa*	1	Positive	NT
21GA0485	Tattnall Co., GA	2021	*Allium cepa*	1	Positive	NT
21GA0486	Tattnall Co., GA	2021	*Allium cepa*	1	Positive	NT
21GA0487	Tattnall Co., GA	2021	*Allium cepa*	1	Positive	NT
21GA0488	Tattnall Co., GA	2021	*Allium cepa*	1	Positive	NT
21GA0490	Tattnall Co., GA	2021	Weed leaf	0	Positive	NT
21GA0531	Toombs Co., GA	2021	*Allium cepa*	1	Positive	NT
21GA0534	Toombs Co., GA	2021	*Allium cepa*	1	Positive	NT
21GA0535	Toombs Co., GA	2021	*Allium cepa*	1	Positive	NT
21GA0536	Toombs Co., GA	2021	*Allium cepa*	1	Positive	NT
21GA0537	Toombs Co., GA	2021	*Allium cepa*	1	Positive	NT
21GA0553	Tattnall Co., GA	2021	*Allium cepa*	1	Positive	NT
21GA0555	Tattnall Co., GA	2021	*Allium cepa*	1	Positive	NT
21GA0556	Tattnall Co., GA	2021	*Allium cepa*	1	Positive	NT
21GA0557	Tattnall Co., GA	2021	*Allium cepa*	1	Positive	NT
21GA0559	Tattnall Co., GA	2021	*Allium cepa*	1	Positive	NT
21GA0560	Tattnall Co., GA	2021	*Allium cepa*	1	Positive	NT
21GA0563	Tattnall Co., GA	2021	*Allium cepa*	0	Positive	NT
21GA0565	Tattnall Co., GA	2021	*Allium cepa*	1	Positive	NT
21GA0567	Tattnall Co., GA	2021	*Allium cepa*	1	Positive	NT
21GA0568	Tattnall Co., GA	2021	*Allium cepa*	1	Positive	NT
21GA0569	Tattnall Co., GA	2021	*Allium cepa*	1	Positive	NT
Pa03_1	Mitchell Co. GA	2003	*Brassica* sp*.*	0	Positive	NT
Pa200_1	Tattnall Co. GA	2000	*Allium cepa*	1	Positive	NT
Pa89_2	Vidalia, GA	1989	*Capsicum annuum*	0	Positive	NT
Pa90_1	Tattnall Co. GA	1990	*Allium cepa*	1	Positive	NT
Pa90_2	Toombs Co. GA	1990	*Allium cepa*	1	Positive	NT
Pa90_3	Toombs Co. GA	1990	*Allium cepa*	1	Positive	NT
Pa90_4	Tattnall Co. GA	1990	*Allium cepa*	1	Positive	NT
Pa90_5	Tattnall Co. GA	1990	*Allium cepa*	1	Positive	NT
Pa90_7	Tattnall Co. GA	1990	*Allium cepa*	1	Positive	NT
Pa91_303	Tift Co. GA	1991	*Allium cepa*	1	Negative	Negative
Pa91_304	Tift Co. GA	1991	*Allium cepa*	1	Negative	Negative
Pa91_305	Tift Co. GA	1991	*Allium cepa*	1	Negative	Negative
Pa91_306	Tift Co. GA	1991	*Allium cepa*	1	Negative	Negative
Pa91_308	Tift Co. GA	1991	*Allium cepa*	1	Negative	Negative
Pa91_309	Tift Co. GA	1991	*Allium cepa*	1	Positive	NT
Pa91_311	Tift Co. GA	1991	*Allium cepa*	1	Negative	Negative
Pa91_312	Tift Co. GA	1991	*Allium cepa*	1	Negative	Negative
Pa91_313	Tift Co. GA	1991	*Allium cepa*	1	Negative	Negative
Pa91_314	Tift Co. GA	1991	*Allium cepa*	0	Negative	Negative
Pa91_315	Tift Co. GA	1991	*Allium cepa*	1	Negative	Negative
Pa91_317	Tift Co. GA	1991	*Allium cepa*	1	Positive	NT
Pa91_318	Tift Co. GA	1991	*Allium cepa*	1	Negative	Negative
Pa91_319	Tift Co. GA	1991	*Allium cepa*	0	Negative	Negative
Pa91_321	Tift Co. GA	1991	*Allium cepa*	1	Negative	Negative
Pa91_322	Tift Co. GA	1991	*Allium cepa*	1	Negative	Negative
Pa91_323	Tift Co. GA	1991	*Allium cepa*	0	Negative	Negative
Pa92_1	Vidalia, GA	1992	*Allium cepa*	0	Positive	NT
Pa93_10	Tattnall Co. GA	1993	Cruciferous weed	0	Positive	NT
Pa93_11	Tattnall Co. GA	1993	Cruciferous weed	0	Positive	NT
Pa93_12	Tattnall Co. GA	1993	Cruciferous weed	0	Positive	NT
Pa93_13	Tattnall Co. GA	1993	Cruciferous weed	0	Positive	NT
Pa93_2	Tattnall Co. GA	1993	Cruciferous weed	1	Positive	NT
Pa93_3	Tattnall Co. GA	1993	Cruciferous weed	0	Positive	NT
Pa93_300	Tattnall Co. GA	1993	Cruciferous weed	0	Positive	NT
Pa93_301	Tattnall Co. GA	1993	Cruciferous weed	0	Positive	NT
Pa93_302	Tattnall Co. GA	1993	Cruciferous weed	0	Positive	NT
Pa93_303	Tattnall Co. GA	1993	Cruciferous weed	0	Positive	NT
Pa93_304	Tattnall Co. GA	1993	Cruciferous weed	0	Positive	NT
Pa93_305	Tattnall Co. GA	1993	Cruciferous weed	0	Positive	NT
Pa93_5	Tattnall Co. GA	1993	Cruciferous weed	0	Positive	NT
Pa93_6	Tattnall Co. GA	1993	Cruciferous weed	0	Positive	NT
Pa93_7	Tattnall Co. GA	1993	Cruciferous weed	0	Positive	NT
Pa93_8	Tattnall Co. GA	1993	Cruciferous weed	1	Positive	NT
Pa93_9	Tattnall Co. GA	1993	Cruciferous weed	0	Positive	NT
Pa95_1	Tift Co. GA	1995	*Allium cepa*	1	Positive	NT
Pa95_3	Tift Co. GA	1995	*Allium cepa*	0	Positive	NT
Pa95_4	Tift Co. GA	1995	*Allium cepa*	0	Positive	NT
Pa95_5	Tift Co. GA	1995	*Allium cepa*	0	Positive	NT
Pa95_7	Vidalia, GA	1995	*Allium cepa*	1	Positive	NT
Pa95_9	Vidalia, GA	1995	*Oenothera laciniata*	0	Positive	NT
Pa98_1	Georgia	1998	*Allium cepa*	1	Positive	NT
Pa98_2	Tift Co. GA	1998	*Allium cepa*	0	Positive	NT
Pa98_3	Bulloch Co, GA	1998	*Allium cepa*	1	Positive	NT
Pa99_1	Tattnall Co. GA	1999	*Allium cepa*	2	Positive	NT
Pa99_5	Toombs Co. GA	1999	*Allium cepa*	1	Positive	NT
Pa99_6	Colquitt Co. GA	1999	*Brassica rapa* subsp. *rapa*	0	Positive	NT

^*a*^ NT, not tested.

### DNA extraction

Genomic DNA extraction from *P. alliivorans* strains was carried out using the Monarch Genomic DNA Purification Kit (New England Biolabs, Ipswich, MA, USA) following the manufacturer’s protocol. DNA concentration and quality were evaluated using a Nanodrop spectrophotometer (Thermo Fisher Scientific, Waltham, MA, USA).

### Initial identification of isolated bacteria

Initial identification of the isolated bacteria was performed in two steps, using genomic DNA extracted as described above. First, we performed 16S rRNA gene sequencing to confirm membership in the genus *Pseudomonas*. The 16S rRNA gene was amplified using universal primers 27F/1492R (18). PCR products were sequenced, and the resulting sequences were compared against the National Center for Biotechnology Information (NCBI) database using BLASTn. Second, for isolates confirmed as *Pseudomonas* spp., we performed citrate synthase (*cts*) housekeeping gene sequence analysis (14). The *cts* gene was amplified via polymerase chain reaction (PCR) with *cts-*specific primers (14). The PCR products were then sequenced. The obtained *cts* sequences were analyzed and compared with known reference sequences (14) to confirm the identity of the isolated *P. alliivorans* strains.

### Genome sequencing and assembly

For genome sequencing, genomic libraries were constructed according to the manufacturer’s instructions using the NEBNext Ultra II DNA Library Prep Kit for Illumina. Sequencing was carried out on the Illumina NovaSeq 6000 platform by Novogene Co., Ltd. (Beijing, China) using paired-end sequencing to generate high-quality reads for subsequent assembly. After sequencing, the paired-end reads underwent initial quality filtering and trimming to remove low-quality bases and adapters using Trimmomatic v0.38. Read quality was assessed using FastQC v0.11.5 (https://www.bioinformatics.babraham.ac.uk/projects/fastqc/). The processed reads were assembled *de novo* using SPAdes v3.14 with the—isolate --cov-cutoff auto mode (19). Contigs shorter than 500 bp in length were removed. The final assemblies were evaluated for completeness and contamination using CheckM (20) and annotated automatically using the NCBI Prokaryotic Genome Annotation Pipeline (21). Plasmid sequences in all 113 *P*. *alliivorans* genomes (110 newly sequenced genomes and three previously published genomes) were predicted using PlasmidHunter v1.3 (22), a tool that predicts plasmids based on gene content profiles. Detailed information on assembly statistics, including plasmid-related contigs and accession numbers for all genome sequence data utilized in this study, can be found in Table 2 and Table S1.

**TABLE 2:** General features of *P. alliivorans* genomes

Strain	dDDH (%) relative to *P. alliivorans* 20GA0068^T^	Total sequence length (bp)	GC	Total gene	Protein-coding gene	N50	Contig	Number of plasmid-related contigs	Completeness	BioProject accession	BioSample accession
20GA0068	100.0	5,839,609	59.14	5,260	5,116	487,261	31	4	100	PRJNA700779	SAMN17838827
20GA0069	100.0	5,840,909	59.14	5,280	5,141	716,644	29	4	100	PRJNA1069880	SAMN39625729
20GA0070	100.0	5,840,694	59.14	5,280	5,142	716,644	30	4	100	PRJNA1069880	SAMN39625730
20GA0080	91.6	5,853,898	59.04	5,324	5,169	433,142	26	3	100	PRJNA700779	SAMN17838832
20GA0081	91.1	5,831,183	59.14	5,268	5,151	784,869	22	3	100	PRJNA1069880	SAMN39625731
20GA0082	91.0	5,851,415	59.09	5,275	5,147	811,451	21	6	100	PRJNA1069880	SAMN39625732
20GA0083	90.7	5,729,121	59.27	5,161	5,041	343,784	44	6	100	PRJNA1069880	SAMN39625733
20GA0084	91.3	5,989,735	58.98	5,418	5,293	681,511	31	6	100	PRJNA1069880	SAMN39625734
20GA0148	90.4	5,982,655	59.01	5,391	5,247	342,249	40	6	100	PRJNA700779	SAMN17838833
20GA0149	90.4	5,983,427	59.01	5,406	5,273	342,249	39	6	100	PRJNA1069880	SAMN39625735
20GA0198	90.4	5,799,328	59.17	5,281	5,138	798,476	23	2	100	PRJNA1069880	SAMN39625736
20GA0201	98.6	5,859,691	59.18	5,338	5,205	523,786	31	6	100	PRJNA1069880	SAMN39625737
20GA0207	91.6	6,007,949	59.10	5,506	5,371	515,387	36	7	100	PRJNA1069880	SAMN39625738
20GA0227	91.1	6,029,914	58.97	5,461	5,336	774,606	29	8	100	PRJNA1069880	SAMN39625739
20GA0228	91.8	5,876,897	59.09	5,331	5,208	598,888	32	7	100	PRJNA1069880	SAMN39625740
20GA0233	91.6	5,966,899	59.08	5,450	5,328	972,100	17	3	100	PRJNA1069880	SAMN39625741
20GA0235	91.9	5,929,610	58.97	5,403	5,248	435,098	40	9	100	PRJNA1069880	SAMN39625742
20GA0237	91.3	5,924,734	59.11	5,359	5,235	556,363	29	3	100	PRJNA1069880	SAMN39625743
21GA0411	91.4	5,943,933	59.02	5,418	5,299	556,518	29	4	100	PRJNA1069880	SAMN39625744
21GA0412	91.4	5,754,198	59.19	5,222	5,098	556,838	28	7	100	PRJNA1069880	SAMN39625745
21GA0416	91.4	5,753,674	59.18	5,226	5,098	423,745	30	7	100	PRJNA1069880	SAMN39625746
21GA0420	91.3	5,883,376	59.11	5,329	5,212	782,168	27	7	100	PRJNA1069880	SAMN39625747
21GA0421	90.8	5,746,990	59.24	5,167	5,053	555,018	29	4	100	PRJNA1069880	SAMN39625748
21GA0425	91.4	5,802,940	59.14	5,249	5,124	545,871	33	3	100	PRJNA1069880	SAMN39625749
21GA0426	91.5	5,846,613	59.09	5,266	5,139	534,077	35	6	100	PRJNA1069880	SAMN39625750
21GA0427	91.6	5,825,648	59.14	5,268	5,144	343,262	42	4	100	PRJNA1069880	SAMN39625751
21GA0475	91.6	5,882,870	59.06	5,384	5,244	359,879	56	7	100	PRJNA1069880	SAMN39625752
21GA0476	91.6	5,772,178	59.20	5,212	5,081	401,654	28	5	100	PRJNA1069880	SAMN39625753
21GA0477	90.6	5,912,753	59.15	5,379	5,248	366,452	27	0	100	PRJNA1069880	SAMN39625754
21GA0478	91.2	5,853,884	59.14	5,254	5,137	936,246	17	3	100	PRJNA1069880	SAMN39625755
21GA0479	90.3	5,746,167	59.26	5,192	5,074	779,078	29	7	100	PRJNA1069880	SAMN39625756
21GA0480	91.5	5,870,521	59.09	5,313	5,192	353,492	43	8	100	PRJNA1069880	SAMN39625757
21GA0481	91.2	5,769,143	59.16	5,207	5,091	305,582	36	4	100	PRJNA1069880	SAMN39625758
21GA0482	91.2	5,770,218	59.16	5,210	5,093	305,582	36	4	100	PRJNA1069880	SAMN39625759
21GA0483	91.2	5,771,150	59.16	5,204	5,087	452,002	32	4	100	PRJNA1069880	SAMN39625760
21GA0484	91.2	5,767,594	59.16	5,201	5,089	423,492	35	4	100	PRJNA1069880	SAMN39625761
21GA0485	90.4	5,981,121	59.01	5,405	5,276	342,249	41	6	100	PRJNA1069880	SAMN39625762
21GA0486	91.2	5,769,466	59.16	5,207	5,090	423,418	33	4	100	PRJNA1069880	SAMN39625763
21GA0487	91.2	5,769,472	59.16	5,201	5,086	314,409	35	4	100	PRJNA1069880	SAMN39625764
21GA0488	91.2	5,769,177	59.16	5,205	5,090	314,409	34	4	100	PRJNA1069880	SAMN39625765
21GA0490	91.4	5,835,180	59.06	5,271	5,132	333,995	48	6	100	PRJNA1069880	SAMN39625766
21GA0531	91.4	5,753,083	59.19	5,224	5,102	147,208	64	8	100	PRJNA1069880	SAMN39625767
21GA0534	91.4	5,752,633	59.19	5,220	5,096	554,892	28	7	100	PRJNA1069880	SAMN39625768
21GA0535	91.4	5,754,584	59.18	5,225	5,097	554,958	31	7	100	PRJNA1069880	SAMN39625769
21GA0536	91.4	5,754,342	59.18	5,226	5,099	556,904	28	7	100	PRJNA1069880	SAMN39625770
21GA0537	91.4	5,753,169	59.19	5,223	5,099	330,041	35	7	100	PRJNA1069880	SAMN39625771
21GA0553	90.4	5,981,153	59.01	5,404	5,276	292,890	47	7	100	PRJNA1069880	SAMN39625772
21GA0555	90.4	5,978,841	59.02	5,409	5,279	190,040	84	13	100	PRJNA1069880	SAMN39625773
21GA0556	90.4	5,980,480	59.01	5,407	5,276	200,372	52	7	100	PRJNA1069880	SAMN39625774
21GA0557	90.4	5,982,089	59.01	5,407	5,276	336,296	47	7	100	PRJNA1069880	SAMN39625775
21GA0559	90.4	5,981,268	59.01	5,406	5,274	342,249	40	6	100	PRJNA1069880	SAMN39625776
21GA0560	90.4	5,981,280	59.01	5,401	5,272	342,249	42	6	100	PRJNA1069880	SAMN39625777
21GA0563	91.2	5,827,188	59.12	5,284	5,164	585,019	28	3	100	PRJNA1069880	SAMN39625778
21GA0565	90.4	5,981,940	59.01	5,407	5,274	292,884	46	7	100	PRJNA1069880	SAMN39625779
21GA0567	89.9	5,897,833	59.09	5,356	5,228	336,522	37	3	100	PRJNA1069880	SAMN39625780
21GA0568	90.4	6,096,400	59.02	5,512	5,377	200,376	55	9	100	PRJNA1069880	SAMN39625781
21GA0569	91.2	5,767,368	59.16	5,208	5,092	193,695	61	6	100	PRJNA1069880	SAMN39625782
Pa03_1	91.6	5,696,142	59.25	5,131	5,005	611,409	23	5	100	PRJNA1069880	SAMN39625783
Pa200_1	91.0	5,783,148	59.18	5,207	5,095	892,536	20	3	99.68	PRJNA1069880	SAMN39625784
Pa89_2	91.1	5,914,032	59.24	5,346	5,232	701,223	32	3	99.68	PRJNA1069880	SAMN39625785
Pa90_1	91.3	5,780,435	59.22	5,245	5,123	566,525	25	3	100	PRJNA1069880	SAMN39625786
Pa90_2	91.4	5,701,126	59.28	5,148	5,028	447,880	28	4	100	PRJNA1069880	SAMN39625787
Pa90_3	91.4	5,702,138	59.28	5,149	5,030	554,169	28	4	100	PRJNA1069880	SAMN39625788
Pa90_4	91.2	5,754,554	59.22	5,186	5,060	774,380	22	4	100	PRJNA1069880	SAMN39625789
Pa90_5	91.4	5,806,289	59.15	5,257	5,131	520,390	32	7	100	PRJNA1069880	SAMN39625790
Pa90_7	91.2	5,754,647	59.22	5,186	5,058	971,105	21	4	100	PRJNA1069880	SAMN39625791
Pa91_303	91.3	5,701,340	59.25	5,147	5,025	969,020	29	5	100	PRJNA1069880	SAMN39625792
Pa91_304	91.3	5,702,566	59.25	5,149	5,027	969,020	28	5	100	PRJNA1069880	SAMN39625793
Pa91_305	91.3	5,702,804	59.25	5,147	5,023	969,020	28	5	100	PRJNA1069880	SAMN39625794
Pa91_306	91.3	5,702,483	59.25	5,152	5,029	554,951	31	6	100	PRJNA1069880	SAMN39625795
Pa91_308	91.3	5,700,294	59.25	5,150	5,026	555,329	32	5	100	PRJNA1069880	SAMN39625796
Pa91_309	91.3	5,702,483	59.25	5,152	5,028	820,843	26	4	100	PRJNA1069880	SAMN39625797
Pa91_311	91.3	5,702,781	59.25	5,152	5,026	969,020	30	6	100	PRJNA1069880	SAMN39625798
Pa91_312	91.3	5,702,617	59.25	5,148	5,025	969,020	26	5	100	PRJNA1069880	SAMN39625799
Pa91_313	91.3	5,701,368	59.25	5,148	5,025	816,059	26	4	100	PRJNA1069880	SAMN39625800
Pa91_314	90.6	5,780,552	59.23	5,227	5,098	767,797	21	4	100	PRJNA1069880	SAMN39625801
Pa91_315	91.3	5,701,564	59.25	5,149	5,024	816,062	29	4	100	PRJNA1069880	SAMN39625802
Pa91_317	91.3	5,701,859	59.25	5,151	5,026	956,587	30	5	100	PRJNA1069880	SAMN39625803
Pa91_318	91.3	5,698,923	59.25	5,150	5,026	497,262	31	5	100	PRJNA1069880	SAMN39625804
Pa91_319	90.6	5,781,492	59.23	5,225	5,097	697,856	21	4	100	PRJNA1069880	SAMN39625805
Pa91_321	90.4	5,936,906	59.06	5,356	5,219	822,792	27	4	100	PRJNA1069880	SAMN39625806
Pa91_322	91.3	5,704,818	59.25	5,152	5,028	577,536	28	4	100	PRJNA1069880	SAMN39625807
Pa91_323	90.6	5,780,360	59.23	5,227	5,098	697,856	22	4	100	PRJNA1069880	SAMN39625808
Pa92_1	89.8	5,778,235	59.21	5,207	5,098	469,893	37	6	100	PRJNA1069880	SAMN39625809
Pa93_10	89.9	5,877,472	59.13	5,311	5,182	551,997	34	9	99.68	PRJNA1069880	SAMN39625810
Pa93_11	91.7	5,825,853	59.16	5,297	5,165	439,821	30	3	100	PRJNA1069880	SAMN39625811
Pa93_12	89.8	5,942,121	59.12	5,410	5,285	376,156	38	5	100	PRJNA1069880	SAMN39625812
Pa93_13	90.3	5,827,713	59.13	5,272	5,154	712,572	25	4	100	PRJNA1069880	SAMN39625813
Pa93_2	91.4	5,894,427	58.99	5,396	5,245	406,146	47	9	100	PRJNA1069880	SAMN39625814
Pa93_3	91.1	5,784,134	59.23	5,230	5,113	403,146	32	6	100	PRJNA1069880	SAMN39625815
Pa93_300	91.6	5,836,744	59.04	5,329	5,181	548,686	42	5	99.68	PRJNA1069880	SAMN39625816
Pa93_301	90.4	5,825,424	59.12	5,269	5,153	777,837	26	5	100	PRJNA1069880	SAMN39625817
Pa93_302	89.8	5,943,913	59.13	5,412	5,285	379,426	37	4	100	PRJNA1069880	SAMN39625818
Pa93_303	89.7	5,944,229	59.13	5,412	5,287	379,426	36	4	100	PRJNA1069880	SAMN39625819
Pa93_304	89.9	5,723,308	59.22	5,143	5,019	524,228	29	5	99.68	PRJNA1069880	SAMN39625820
Pa93_305	90.0	5,722,374	59.22	5,146	5,021	551,242	29	5	99.68	PRJNA1069880	SAMN39625821
Pa93_5	90.3	5,825,097	59.12	5,276	5,157	712,572	29	4	100	PRJNA1069880	SAMN39625822
Pa93_6	91.6	5,839,411	59.04	5,332	5,182	555,325	41	8	99.68	PRJNA1069880	SAMN39625823
Pa93_7	90.0	5,722,935	59.22	5,142	5,019	551,242	29	5	99.68	PRJNA1069880	SAMN39625824
Pa93_8	91.3	5,708,802	59.28	5,174	5,057	575,326	27	6	100	PRJNA1069880	SAMN39625825
Pa93_9	90.3	5,820,604	59.12	5,270	5,152	553,643	29	6	100	PRJNA1069880	SAMN39625826
Pa95_1	89.2	5,964,868	59.05	5,387	5,267	808,605	23	3	100	PRJNA1069880	SAMN39625827
Pa95_3	89.9	5,721,427	59.22	5,141	5,016	524,228	28	5	99.68	PRJNA1069880	SAMN39625828
Pa95_4	90.0	5,722,120	59.22	5,141	5,017	551,242	28	5	99.68	PRJNA1069880	SAMN39625829
Pa95_5	89.9	5,723,474	59.22	5,146	5,021	551,248	30	5	99.68	PRJNA1069880	SAMN39625830
Pa95_7	90.6	5,892,248	59.10	5,335	5,208	847,613	22	3	100	PRJNA1069880	SAMN39625831
Pa95_9	91.5	5,824,816	59.16	5,287	5,165	782,200	25	4	100	PRJNA1069880	SAMN39625832
Pa98_1	91.5	5,727,512	59.22	5,191	5,071	681,362	25	2	100	PRJNA1069880	SAMN39625833
Pa98_2	91.4	5,881,003	59.08	5,313	5,186	593,600	28	1	100	PRJNA1069880	SAMN39625834
Pa98_3	90.3	5,987,657	59.08	5,450	5,302	612,769	16	0	100	PRJNA1069880	SAMN39625835
Pa99_1	90.6	5,908,954	59.05	5,354	5,229	551,774	27	4	100	PRJNA1069880	SAMN39625836
Pa99_5	91.8	5,930,872	58.98	5,403	5,243	604,272	24	4	100	PRJNA1069880	SAMN39625837
Pa99_6	91.4	5,795,508	59.16	5,273	5,148	457,950	35	5	100	PRJNA1069880	SAMN39625838

### Digital DNA-DNA hybridization (dDDH) and average nucleotide identity (ANI)

The assemblies were analyzed using the TYGS web server for digital DNA-DNA hybridization (dDDH) (23). Average nucleotide identity (ANI) values were calculated using pyani v0.2.12 with default parameters and the ANIb method (24) against the whole genome of the *P. alliivorans* type strain 20GA0068^T^.

### Pan-genome and core-proteome analysis

The draft genomes of the 113 *P*. *alliivorans* strains (110 newly sequenced genomes and three previously published genomes) were annotated using Prokka v1.14.5 (25). The resulting GFF3 files were used as input in Roary v3.13.0 (26) to calculate the pan-genome.

For the whole-proteome analysis, the (.faa) annotated protein files were used as input in OrthoFinder v2.5.4 (27, 28) to identify orthogroups. Unrooted gene trees were constructed from these orthogroups employing the DendroBLAST algorithm (29). Subsequently, the STAG algorithm inferred an unrooted species tree from a set of unrooted gene trees (30). This species tree was then rooted using the STRIDE algorithm, which infers the root position based on patterns of well-supported gene duplication events (31). The final cladogram was visualized with the R package ggtree (32).

We used ClonalFrameML v1.13 (33) to investigate homologous recombination among 115 *Pseudomonas alliivorans* genomes (113 genomes from strains isolated in Georgia and two publicly available genomes) and several closely related *Pseudomonas* species. Genomes were annotated using Prokka v1.14.5 to generate GFF3 files, from which a core-genome alignment was produced using Roary v3.13.0. A maximum likelihood phylogeny under the GTR+GAMMA substitution model was inferred with RAxMLv12.2.0 and used as the input guide tree. ClonalFrameML infers homologous recombination by first reconstructing ancestral states on a given maximum‐likelihood phylogeny, then applying a hidden Markov model coupled with an Expectation-Maximization algorithm to estimate recombination parameters (R/θ, δ, and ν) and locate recombination tracts on each branch of the tree. The recombination-aware phylogeny and genome-wide recombination tract mapping were visualized in RStudio. Type, pathotype, and representative strains from the *P. syringae* species complex were selected based on previous reports (14, 34, 35).

### Pathogenicity assays

The pathogenicity of each bacterial strain on onions was assessed using two assays: a foliar assay and a red onion scale assay.

In the foliar assay, following the method previously described (36), onion seedlings (cv. Century) were grown in plastic pots with commercial potting mix in a greenhouse at approximately 25°C. Eight-week-old seedlings were inoculated by cutting the foliage 1 cm from the apex using sterilized scissors. A 10 µL bacterial suspension containing 1 × 10^8^ CFU/mL (~1 × 10^6^ CFU/foliage) was applied twice to the cut end of the foliage. Positive and negative controls consisted of seedlings inoculated with *P. ananatis* PNA97-1R (a known onion pathogen) and sterile water, respectively. Symptom development was assessed at 5 days post-inoculation (dpi), and the experiments were repeated twice with three replicates each.

For the red onion scale assay, red onion bulbs were sliced to expose the fleshy scales. Bacterial strains were cultured overnight in Luria-Bertani (LB) medium at 28°C, then harvested by centrifugation and adjusted to a concentration of 1 × 10^8^ CFU/mL. The bacterial suspension was inoculated onto the onion scales, with sterile water used as the negative control. Following inoculation, the onion scales were incubated for 3 days, and symptoms were evaluated at 3 dpi to determine the pathogenicity of each strain. The red onion scale assays were conducted twice with at least three replicates each.

### Hypersensitivity reaction to tobacco

To assess hypersensitivity reactions (HR) or cell death in tobacco leaves induced by the 15 onion-pathogenicity-negative *P. alliivorans* strains, bacterial strains were cultured overnight in LB medium at 28°C, harvested by centrifugation, and resuspended in 10 mM MgCl_2_ to a final concentration of 10^8^ CFU/mL. Fully expanded leaves of 6- to 8-week-old tobacco plants were infiltrated with 100 µL of the bacterial suspension using a needleless syringe. Sterile 10 mM MgCl_2_ was used as a negative control, and *P. alliivorans* 20GA0068^T^ was included as a positive control. The plants were maintained in a growth chamber at 25°C with a 16 h light/8 h dark cycle. HR symptoms (visible tissue collapse or necrosis) were evaluated at 2 dpi. The experiment was conducted twice with three replicates each.

### Virulence gene prediction

Protein secretion systems were predicted using TXSScan (37, 38). The presence or absence of T-PAI (tripartite pathogenicity island) or S-PAI (single pathogenicity island) was determined by detecting specific genetic markers, namely *hopA* and *shcA in silico*. Both T-PAI and S-PAI encode components of the type III secretion system (T3SS), which is critical for delivering effector proteins into host cells to promote pathogenicity (39, 40). T-PAI is a tripartite structure containing the *hrp/hrc* gene cluster (core T3SS machinery), an exchangeable effector locus, and a conserved effector locus (41). In contrast, S-PAI lacks these additional loci and contains only the *hrp/hrc* cluster with a 10 kb insertion harboring the effector gene *avrE* and its chaperone (39–41). This simplified structure of S-PAI distinguishes it from the more complex T-PAI, which is found in other *Pseudomonas* species such as *P. syringae* pv. *tomato* DC3000 (41). The distribution of T3SS loci was analyzed by conducting BLASTP searches of the type III effectors (T3Es) on http://pseudomonas-syringae.org/ against the 113 *P*. *alliivorans* proteomes. The *alt* sequences were identified using BLASTn. The *alt* sequences from *P. alliivorans* and other *Pseudomonas* spp. were aligned using MAFFT v7.388 (42). A neighbor-joining phylogenetic tree based on the alignment was constructed using Geneious, with the topology’s robustness estimated using 1,000 bootstrap replicates.

### Functional characterization of virulence factors

Mutant strains deficient in specific virulence factors, including T3SSs (*hrcV*), T2SSs (*gspG*), and the *alt* cluster, were created in *P. alliivorans* 20GA0068. The mutants were constructed according to the protocol previously described (43), using the primers and synthesized dsDNA listed in Tables S2 and S3. The Gateway-compatible vector pDONR1K18ms (Addgene plasmid #72644) was used for creating deletion constructs.

To complement the 20GA0068*ΔhrcV* mutant, the *hrcV* gene from 20GA0068^T^ was synthesized with flanking attL recombination sites (Twist Biosciences, San Francisco, CA, USA) (Table S3). The gene was inserted into the expression vector pBS46 carrying attR sites through LR recombination using Gateway LR Clonase II Enzyme mix (Invitrogen, Waltham, MA, USA). The resulting construct (pBS46::*hrcV*) was introduced into *E. coli* MaH1 (44) by electroporation, and the plasmid was subsequently isolated using the GeneJET Plasmid Miniprep Kit (Thermo Fisher Scientific, Waltham, MA, USA). Proper insertion of the *hrcV* fragment into pBS46 was confirmed by PCR using the M13 primer and verified by Sanger DNA sequencing. The validated plasmid was electroporated into electrocompetent 20GA0068Δ*hrcV* cells. Transformants were selected on LB agar supplemented with gentamicin at a concentration of 15 µg/mL. The 20GA0068Δ*hrcV* background was reconfirmed using the *hrcV*-out primer pair (0068hrcVoutF/0068hrcVoutR), and successful insertion of the pBS46::*hrcV* was verified using M13 primers. Primers used to confirm the complementation are listed in Table S2. Wild-type, mutant, and complemented strains were subject to the red onion scale assay as described in the previous section. The average necrosis area was calculated for each inoculated onion scale. Photographs of the scales were taken at 4 dpi. Lesion areas were measured using ImageJ (https://ij.imjoy.io/) (45), calibrated against a known standard scale.

### Zone of inhibition assays

Allicin was prepared according to the method previously outlined (46), with the concentration used being double that described by Stice et al. (46). *P. alliivorans* strains 20GA0068 wild-type strain and *alt* cluster derivative 20GA0068Δ*alt* were streaked for isolation on LB plates supplemented with rifampicin (40 µg/mL). Single colonies from these plates were used to inoculate 5 mL of LB supplemented with rifampicin and incubated with shaking at 30°C overnight. Next, 300 µL of the overnight cultures was spread onto 20 mL LB plates to create a lawn. Once the plates were dry, a well was made in the center of the agar using a sterile 10 µl pipette tip. Synthesized allicin (50 µL) was then added to the well, and the plates were incubated at 30°C for 24 h. To calculate the ZOI area, five points around the circumference of the circle were marked, and the radius of the circle was measured from the center of the well to each of these points. The average radius from the five points was then used to calculate the area of the circle. Each experiment included three technical repeats, and data from three experimental repeats were analyzed for statistical significance using a *t*-test in RStudio.

## RESULTS

### Genome features

In the data set of 113 *P*. *alliivorans* genomes (110 newly sequenced strains and three previously published genomes), the average total sequence length is 5,826,377 base pairs, with an average of 5,272 total genes per strain (Table 2). The average number of protein-coding genes is 5,146. Additionally, the average N50 value, which indicates sequence contiguity, is 561,962 base pairs. The GC content across all 113 strains ranges from 58.97% to 59.28%, with an average of 59.15%. The number of contigs per genome ranged from 16 to 84, with an average of 32. Plasmid-related contigs were identified in 98.2% of strains (111/113), with 1–13 contigs per genome (Table 2). Additionally, completeness scores from the CheckM analysis range from 99.68% to 100%, with an average of 99.97%.

Upon closer examination of individual strains, 21GA0568 has the highest total sequence length, measuring 6,096,400 base pairs. This strain has the largest total gene count of 5,512, along with the highest count of 5,377 protein-coding genes. Moreover, strain 20GA0233 has the highest N50 value at 972,100 base pairs. In contrast, strain Pa03_1 has the smallest total sequence length among *P. alliivorans* strains, measuring 5,696,142 base pairs. This strain also has the lowest total gene number of 5,131 and the smallest number of protein-coding genes at 5,005. Notably, the smallest N50 value in the data set is associated with strain 21GA0531, measured at 147,208 base pairs (Table 1).

### Identification of the strains

To confirm the taxonomic identity of the 110 newly sequenced strains, we employed two genome-scale metrics: Average Nucleotide Identity (ANI) and digital DNA-DNA hybridization (dDDH). For ANI analysis, the lowest pairwise ANI value observed was 98.4% (between Pa99_5 and Pa93_10), which exceeds the 95% threshold commonly used for species delineation (Table S3). For dDDH analysis, the lowest value was 89.2% (between strain Pa95_1 and *P. alliivorans* type strain 20GA0068^T^), surpassing the 70% threshold for species delineation (Table 1). Together, these results conclusively identify all 110 newly sequenced strains as *P. alliivorans*.

Of the 113 *P*. *alliivorans* strains, 71 were isolated from onions, 36 from weeds, two from *Brassica* spp., and one from *Capsicum annuum* (Table 1). Among the weed samples, one specimen was identified as *Oenothera laciniata*, six were sourced from Carolina geranium (*Geranium carolinianum*), and seventeen were from Cruciferous weeds. The remaining weed samples were not further taxonomically classified.

### Pan-genome analysis

To characterize the gene pool diversity of *P. alliivorans*, we analyzed the pan-genome of the 113 strains. The pan-genome consisted of a core genome of 4,552 genes (present in 99%–100% of analyzed genomes, i.e., shared by 111–113 genomes), a soft-core genome of 51 genes (present in 95%–99% of the genomes analyzed, shared by 107–111 genomes), a shell genome of 864 genes (present in 15%–95% of the genomes analyzed, present in 16–107 genomes), and a cloud genome of 8,342 genes (present in <15% of the genomes analyzed, found in <16 genomes), culminating in a total of 13,809 genes (Fig. S1A). Detailed contributions of core and accessory genes from each strain are illustrated in Fig. S1B. Additionally, Fig. S1 visually represents the presence and absence of genes within the pan-genome, highlighting the genomic differences in accessory components and the homogeneity of the core genome across the strains.

### Phylogenetic analysis

To investigate the evolutionary relationships among *P. alliivorans* strains, we first used OrthoFinder to analyze the whole proteomes of the 115 strains (113 Georgia strains + two publicly available *P. alliivorans* genomes, totaling 115 strains) and several closely related *Pseudomonas* species. The OrthoFinder analysis assigned 99.1% of total genes (879,744) to 13,854 orthogroups. Of these, 1,663 orthogroups were shared across all genomes, with 1,068 consisting entirely of single-copy orthologous protein-coding genes.

**Fig 1:**
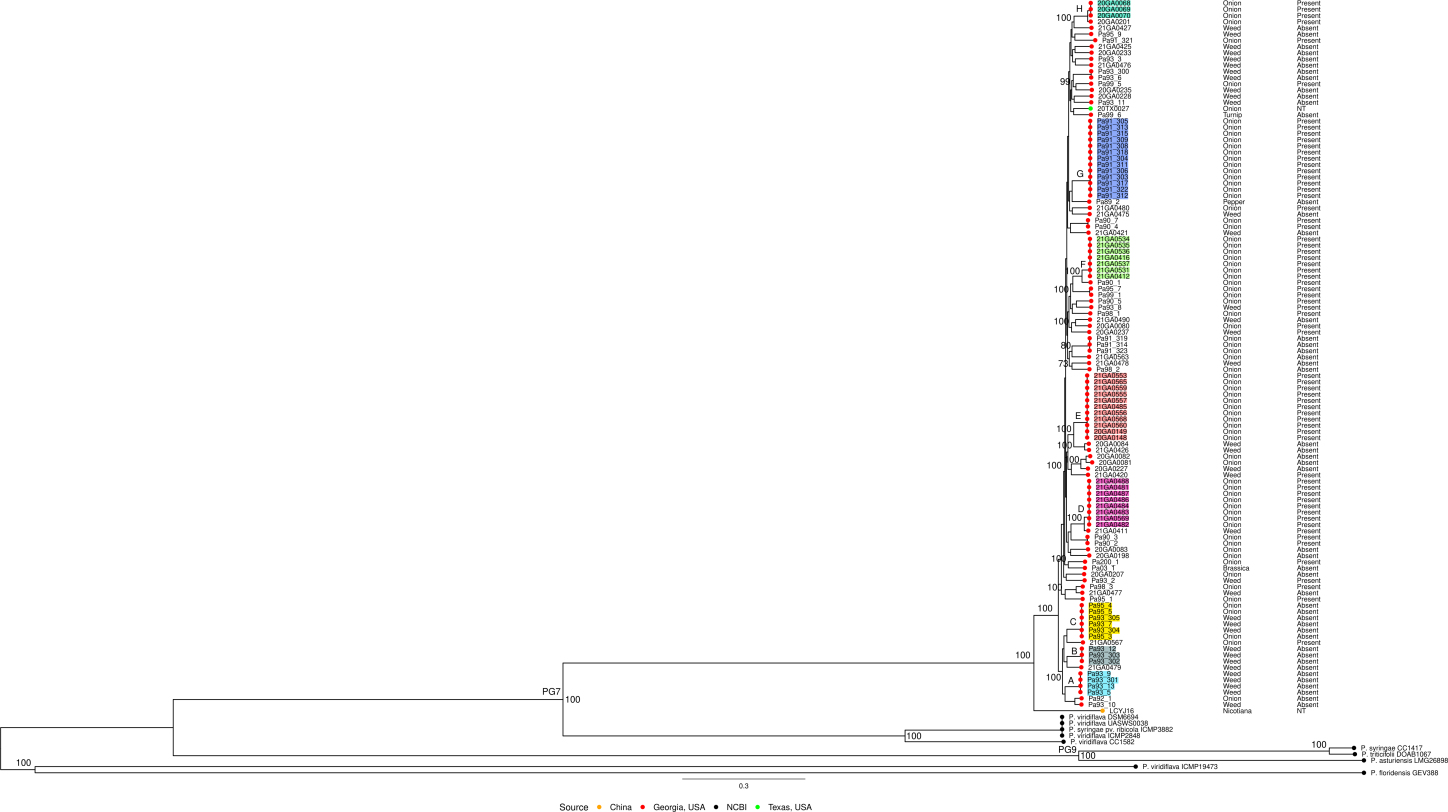
Maximum likelihood phylogeny based on core genome of 125 *Pseudomonas* strains. The phylogenetic tree was inferred using RaxML and drawn with R package ggtree. Tip point colors indicate strain origin: red denotes *P. alliivorans* strains isolated in Georgia (USA) from this study; green represents a *P. alliivorans* strain from Texas (USA); orange indicates a strain isolated in China; and black denotes representative, pathotype, or type strains of *Pseudomonas* species included for reference. Bootstrap values are shown in black along the branches of the tree. PG# represents *Pseudomonas syringae* species complex phylogroups designations. Strains highlighted in A: cyan, B: gray, C: yellow, D: pink, E: orange, F: light green, G: blue, and H: dark green display short branch lengths within the subclade, indicating high genetic similarity.

Phylogenetic reconstructions based on core genes (RaxML) and single-copy orthologous protein-coding genes (OrthoFinder) showed that all 115 *P*. *alliivorans* strains clustered within a single subclade among species of phylogroup 7 (Fig. 1 and Fig. S2). Among them, strain LCYJ16, isolated from *Nicotiana tabacum* in Yunnan, China, was the most divergent. This strain formed a distinct branch at the base of the *P. alliivorans* subclade, indicating its genetic divergence from the other strains included in this study (Fig. 1 and Fig. S2). We observed eight subclades (highlighted as A: cyan, B: gray, C: yellow, D: pink, E: orange, F: light green, G: blue, and H: dark green in Fig. 1 and Fig. S2) within the *P. alliivorans* phylogeny that display highly similar genome content, as indicated by the short branch lengths shared among these strains.

We further used ClonalFrameML to infer homologous recombination events across the core genome alignment of the 115 *P*. *alliivorans* strains. Overall, 2,344 recombination events were detected among the 53 nodes represented by *P. alliivorans* strains. The length of inferred recombination tracts ranged up to 4,646 bp in the genome of strain LCYJ16 (positions 562208–566854), although very short inferred events (i.e., 1 bp in the genome of strain Pa91_317 [positions 1901853–1901854]) may instead represent homoplasy rather than bona fide recombination. ClonalFrameML analysis yielded a posterior mean R/θ  =   0.0905, indicating recombination occurs at nearly one-tenth the rate of point mutations. The estimated inverse mean fragment length (1/δ) was 0.00869, corresponding to a mean imported fragment length (δ = 115 bp), demonstrating that recombination tracts are generally short. The average divergence of imported segments was ν = 0.0315, suggesting donor DNA diverged by approximately 3.1% from recipient genomes.

**Fig 2:**
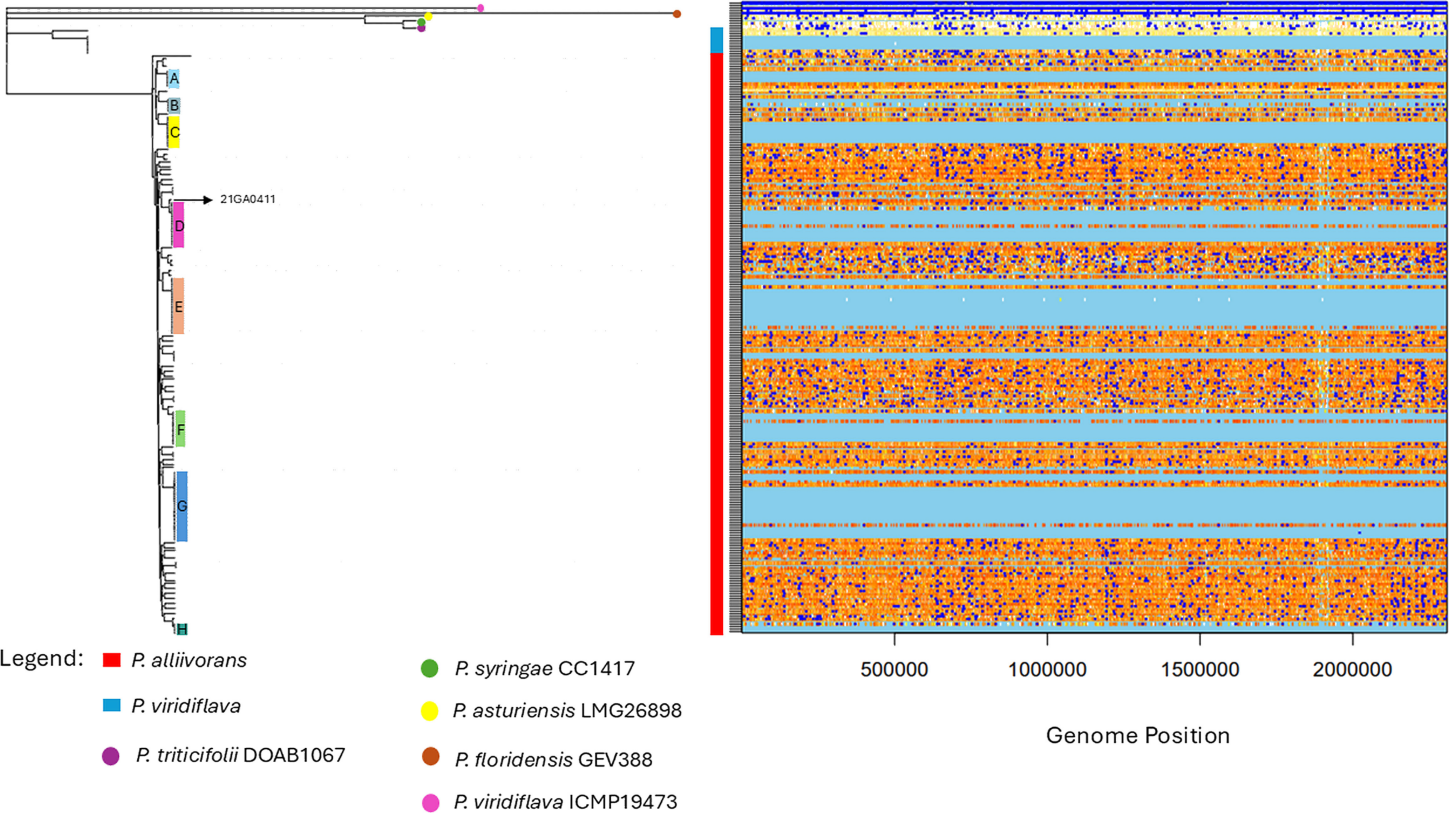
Genome-wide recombination plot and core-genome-based phylogenetic tree (obtained with ClonalFrameML) for the *P. alliivorans* strains and representative closely related species. The plot on the right illustrates recombination patterns among the genomes. Dark blue horizontal bars indicate regions of recombination, whereas light blue areas represent sites that do not change along a given branch. Sites that change are colored based on their level of homoplasy: white indicates no homoplasy, and the gradient from yellow to orange to red reflects increasing degrees of homoplasy. In the phylogenetic tree on the left, red = *P. alliivorans*, blue = *P. viridiflava,* purple = *P. triticifolii,* green = *P. syringae,* yellow = *P. asturiensis,* brown *= P. floridensis,* and pink = *P. viridiflava* ICMP19473. Strains highlighted A: cyan, B: gray, C: yellow, D: pink, E: orange, F: light green, G: blue, and H: dark green display short branch lengths within the subclade, indicating high genetic similarity following the same pattern presented in Fig. 1.

In the recombination-corrected phylogeny, we identified eight clusters of closely related *P. alliivorans* strains that were highlighted as extended light blue segments along plotted branches. According to ClonalFrameML, light blue regions represent genomic windows lacking both point mutations and recombination events, indicating that those strains share highly conserved, non-recombined core genome regions (Fig. 2).

### Pathogenicity in onion

Out of the 113 *P*. *alliivorans* strains examined, 98 strains were pathogenic both on red onion scales and on onion foliage (Table 1). These strains included 59 isolates from onion samples, 36 from weedy plant species, two from cultivated *Brassica* spp. (e.g., cabbage), and one from *Capsicum annuum* (strain Pa89_2, co-isolated from symptomatic pepper leaves with *Pseudomonas syringae* pv. *syringae* and confirmed to be pathogenic on pepper). Notably, upon re-isolation from the lesions, the bacterium displayed colony morphology identical to the initially inoculated strains. It is noteworthy that all strains derived from weed samples exhibited pathogenic phenotypes on onion tissues tested, indicating a critical role for weeds as reservoirs for the dissemination of onion pathogens. Conversely, 15 *P. alliivorans* strains were negative on both red onion scales and on onion foliage (Table 1) and did not elicit an HR on tobacco. Intriguingly, all 15 of these strains were isolated from onions in Tift Co., Georgia, USA in 1991 (Table 1).

### Virulence factors in *P. alliivorans*

#### Allicin tolerance (*alt*) cluster

**Fig 3:**
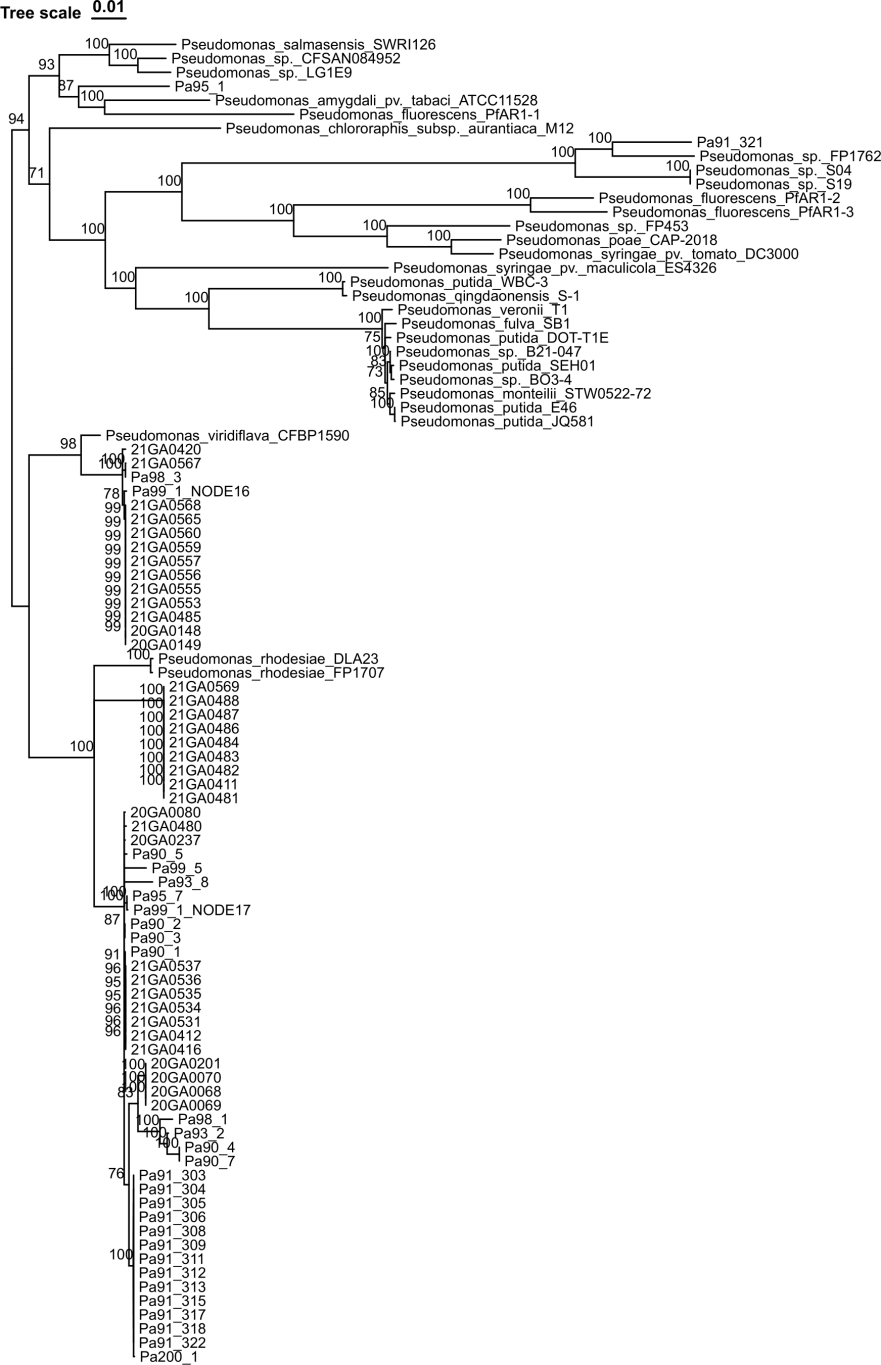
A neighbor-joining phylogenetic tree based on 7,446 bp sequences of the thiosulfinate (allicin)-tolerance (*alt*) gene cluster from *P. alliivorans* and closely related *Pseudomonas* species. The tree was constructed using *alt* cluster sequences from *P. alliivorans* strains isolated from onions and weeds in Georgia, USA, as well as reference *alt* cluster sequences from other *Pseudomonas* species (e.g., *P. fluorescens*, *P. amygdali* pv. *tabaci*, *P. syringae* pv. *tomato*). The tip labels for the strain names of all *P. alliivorans* are displayed in red. Bootstrap values (from 1,000 replicates) are indicated at branch nodes to assess topology robustness. Clustering patterns reveal evolutionary relationships among *alt* clusters, with distinct clades highlighting potential horizontal gene transfer events from diverse donor organisms.

The thiosulfinates tolerance gene or *alt* clusters in onion pathogenic bacterial species *P. ananatis* and *Burkholderia gladioli* pv. *alliicola* confer allicin tolerance *in vitro*. This cluster contributes to red scale necrosis area and associated bacterial colonization on scales in *P. ananatis,* whereas in *Burkholderia gladioli* pv. *alliicola*, it contributes to necrotic length of onion foliage and associated bacterial colonization (46, 47). In the data set of 113 *P. alliivorans* genomes, 65 strains (58% of the total) were identified as containing *alt* clusters. Notably, the majority of these *alt* cluster-positive strains, specifically 60 out of 65 (92%), were isolated from onion samples (Table 1). The remaining five strains were sourced from weed samples. Among the strains with *alt* clusters, 64 had a single *alt* cluster, except for strain Pa99_1, which harbored two distinct *alt* clusters (Pa99_1_NODE16 and Pa99_1_NODE17). These two *alt* cluster sequences from strain Pa99_1 shared 93% pairwise identity out of 7,446 bp aligned sequences and were clustered into separate clades (Fig. 3). Additionally, only one of the 66 *alt* clusters (Pa99_1_NODE16) was localized to a plasmid-related contig, whereas all other *alt* clusters, including Pa99_1_NODE17, were found on chromosomal contigs.

Further analysis based on a neighbor-joining tree of *alt* clusters from *Pseudomonas* spp. revealed that the *alt* cluster from Pa95_1 was more closely related to the group containing *P. fluorescens* PfAR1-1 and *P. amygdali* pv. *tabaci* ATCC 11528. On the other hand, the *alt* cluster from Pa91_321 showed greater similarity to the group containing *P. fluorescens* PfAR1-2 and *P. syringae* pv. *tomato* DC3000. These clusters were distant from all other *P. alliivorans alt* clusters (Fig. 3). The average GC content of all 66 *alt* clusters (including two distinct clusters in strain Pa99_1) across the 113 *P*. *alliivorans* strains is 54.2%, substantially lower than the overall genomic GC content of *P. alliivorans* (59.1%), a hallmark of horizontally acquired DNA. The presence of adjacent IS66 family transposases further supports horizontal gene transfer (HGT) as a potential key mechanism for *alt* cluster acquisition in *P. alliivorans*.

**Fig 4:**
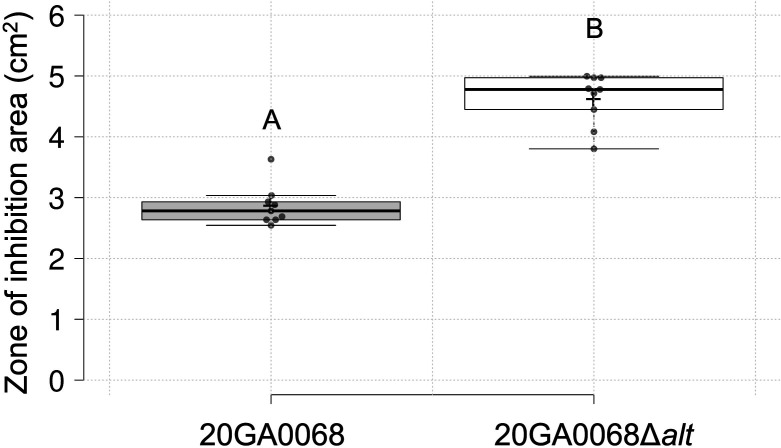
The thiosulfinate tolerance *alt* cluster contributes to allicin tolerance in *P. alliivorans* strain 20GA0068. A box plot graph comparing the allicin zone of inhibition area between strains 20GA0068 and 20GA0068Δ*alt* from three experimental repeats is shown. The center lines represent the medians, the box limits indicate the 25th and 75th percentiles as determined by R software, and the whiskers extend 1.5 times the interquartile range from the 25th and 75th percentiles. Outliers are represented by dots, crosses indicate sample means, and data points are plotted as circles. There is a total of nine sample points (*n* = 9). Different letters indicate significant differences (*P*  <  0.01) between treatments according to the *t*-test.

**Fig 5:**
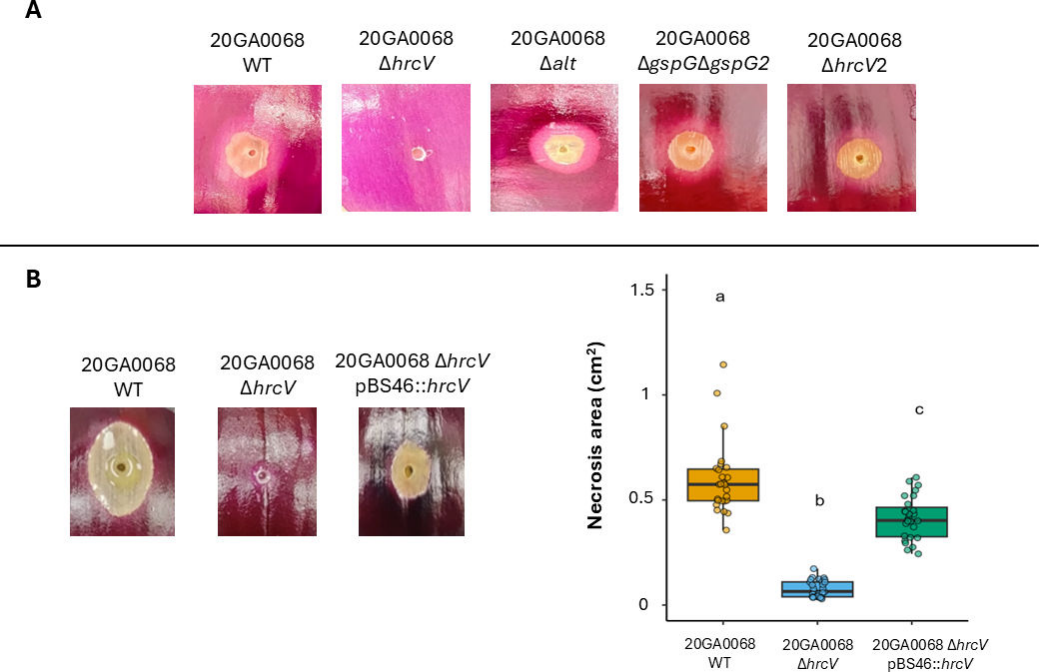
Assessment of virulence factors in *P. alliivorans* on onion scale. The WT = wild-type (*P. alliivorans* 20GA0068) and its deletion mutant derivatives, Δ*hrcV*, Δ*hrcV* pBS46::*hrcV = hrcV* complemented strain, Δ*hrcV*2, T2SSs (Δ*gspG*Δ*gspG2*), and *alt* cluster (Δ*alt*) were assessed for their potential to cause necrosis on the red onion scale. (**A**) Phenotypes produced by mutant strains at 3 days post-inoculation (dpi) on red onion scales, following inoculation with 1 × 10^6^ CFU. (**B**) The figure represents phenotypes of the *hrcV* mutant and complemented strains, along with box plots of their mean necrosis area on red onion scales. The red scale phenotypes shown are representative of three replicates for each treatment (WT, mutant derivatives, and a complemented strain of *hrcV*) across three independent experiments. For the box plot, different letters indicate significant differences (*P* < 0.05) among treatments, according to Tukey-Kramer’s honest significant difference test.

In order to investigate the role of the *alt* cluster in *P. alliivorans* strain 20GA0068^T^, a deletion mutant, 20GA0068Δ*alt*, was created, which lacks the entire *alt* cluster. *In vitro* experiments revealed a significantly larger ZOI in the *P. alliivorans* strain 20GA0068Δ*alt* compared with the wild-type strain 20GA0068^T^ (*P* < 0.01) (Fig. 4). However, assessments using red onion scale assays showed that the deletion of the *alt* gene cluster did not impact symptom development (Fig. 5).

#### Protein secretion

Upon examination using the TXSScan tool, multiple secretion systems were identified within the *P. alliivorans* bacterial genomes under examination. Specifically, the tool detected two clusters of the Type 2 Secretion System, two clusters of the T3SS, one cluster of the Type 4a Pilus, three clusters of the Type 5a Secretion System, one cluster of the Type 5b Secretion System, one cluster of the Type 6 Secretion System, and one cluster of Tight Adherence. The presence of these diverse secretion systems suggests the adaptive capabilities of *P. alliivorans* to various environments.

#### T2SS

To examine the function of the T2SS in *P. alliivorans* strain 20GA0068^T^, a gene deletion mutant (20GA0068Δ*gspG*Δ*gspG2*) lacking both *gspG* genes in the two T2SS clusters was generated. The double mutant strain 20GA0068Δ*gspG*Δ*gspG2* showed necrosis levels similar to the wild-type strain in the red onion scales assay (Fig. 5). This finding suggests that the presence of both T2SSs does not play a noticeable role in inducing symptom development by *P. alliivorans* on red onion scales.

#### T3SSs

The T3SS is a complex nanomachine made up of approximately 25 proteins and has diversified into seven distinct families: Ysc, Hrp1, Hrp2, SPI-1, SPI-2, Rhizobiaceae, and Chlamydia. In the genomes of *P. alliivorans*, two T3SS clusters were identified. The first cluster, referred to as T3SS-1, closely resembles the Hrp1 family of T3SS found in pathogenic *P. syringae* strains, consisting of 29 genes. The second putative T3SS, termed T3SS-2, comprises 17 genes and exhibits great similarity to the rhizobial-like T3SS Rhc of the Rhizobiales family. Interestingly, investigation into a mutant strain defective in the rhizobial-like T3SS Rhc (20GA0068Δ*hrcV2*) showed no discernible differences compared with the wild-type strain (20GA0068) on red onion scales (Fig. 5). However, the mutant strain 20GA0068Δ*hrcV* with the *hrcV* gene deleted from Hrp1-T3SS did not induce any symptom development in the red onion scale assay. Complementation of 20GA0068Δ*hrcV* with the wild-type *hrcV* gene (strain 20GA0068Δ*hrcV* pBS46::*hrcV*) restored necrosis to levels comparable with the wild-type strain, confirming that the loss of pathogenicity in 20GA0068Δ*hrcV* was specifically due to the deletion of *hrcV* and validating the critical role of Hrp1-T3SS in *P. alliivorans* virulence on red onion scales (Fig. 5).

#### T3Es

The analysis of 113 *P. alliivorans* genomes revealed that all of them contained the *avrE* and *hopB/hopAC* effector genes. The *hopBK* gene in nine strains (Pa90_1, Pa95_7, Pa95_9, 20GA0082, 20GA0207, 20GA0235, 21GA0420, 21GA0477, and 21GA0567) was found to be highly similar in sequence. In contrast to closely related *P. viridiflava* strains, which have *hopA* effectors integrated into the T-PAI T3SS structure (39), none of the examined *P. alliivorans* genomes possessed *hopA* homologs. This suggests that *P. alliivorans* strains exclusively utilize the S-PAI T3SS structure, characterized by a simplified *hrp/hrc* cluster containing *avrE* (39, 40)*,* with the *avrE* effector located within this island.

## DISCUSSION

In this study, we sequenced *P. alliivorans* genomes isolated from onion, weeds, *Brassica* spp., and *C. annuum* in Georgia, USA. The acquisition of this large collection of *P. alliivorans* strains (110 genomes reported in this study, and three genomes reported in a previous study [7]) enabled us to use genomic approaches to further characterize their phylogeny and diversity. We analyzed the genetic diversity of *P. alliivorans* and also investigated the contribution of specific genes to virulence using reverse genetics. Additionally, we compared *P. alliivorans* populations in Georgia, USA, and examined potential reservoirs for pathogenic strains. Our results provide insights into the diversity, evolution, and transmission of these plant pathogenic bacteria. The identification of a conserved core genome among *P. alliivorans* strains from various agricultural regions in Georgia, USA, highlights the genetic stability and shared ancestry within this microbial population. The conserved core genome suggests a stable set of genes that may facilitate the species’ adaptation and ecological fitness within the farmscapes of Georgia, USA.

Although *P. alliivorans* was only recently described as a named species based on strains isolated from symptomatic onion foliage in Georgia, USA (7), our genomic sequencing results reveal that it has been present in the state for many years, with strains dating back to at least 1990. For our analysis, we included one publicly available *P. alliivorans* genome: strain LCYJ16, isolated in 2020 from *Nicotiana tabacum* in Yunnan, China (GenBank assembly accession GCA_024584185.1). Phylogenetic analysis of core genomes and single-copy orthologous proteins showed that LCYJ16 is the most genetically divergent strain in our data set, forming a distinct basal branch within the *P. alliivorans* clade. *P. alliivorans* has also been reported to infect onions in Texas (48) and cucurbits (watermelon and cucumber) in Florida and Alabama (35). Notably, the strain ICMP 8820 isolated from peach (*Prunus persica*) and currently annotated as *P. viridiflava* in the NCBI database is formally classified as *P. alliivorans* in the Genome Taxonomy Database. Additionally, a newly released public strain isolated from a tomato in Costa Rica in 2021 further expands the documented distribution and host range of *P. alliivorans*, although it was not included in our phylogenetic analyses. This strain nonetheless presents a valuable resource for future studies of the *P. alliivorans* lineage relatedness. Collectively, these observations confirm that *P. alliivorans* infects a broad range of economically important crops, including tomato, tobacco, peach, cucurbits, onion, *Brassica* spp., and pepper. This broad host and geographic scope underscores the need for further research into the pathogenic potential of diverse *P. alliivorans* strains, their role in disease outbreaks, and their capacity to colonize multiple host plants.

Our findings suggest a nuanced pattern in which *P. alliivorans* exhibits both generalist capabilities (with some strains infecting multiple hosts) and evidence of host-associated adaptation, traits shared with many members of the *P. syringae* species complex (12, 17, 35). Genomic and pathogenicity data confirm that individual *P. alliivorans* strains can infect multiple plant species. For example, strain Pa89_2, isolated from symptomatic *C. annuum* (pepper), was pathogenic on onion in our assays, demonstrating cross-host infectivity. Critically, all 36 strains isolated from asymptomatic weeds (e.g., *Oenothera laciniata, G. carolinianum*) were pathogenic on onion foliage and red onion scales, mirroring the virulence of strains directly isolated from onion. Phylogenetic analysis further supports this hypothesis: weed-derived strains such as Pa93_304 (isolated from a weed in 1993) cluster closely with onion-derived strains like Pa95_5 (isolated from an onion in 1995) in the core-proteome tree (Fig. 1), with high ANI values (>98.4%) indicating minimal genomic divergence. Notably, neither Pa93_304 nor Pa95_5 carries the *alt* gene cluster, which is consistent with the lower prevalence of this cluster in weed-associated strains (only 5/36 weed strains vs. 60/65 onion strains). This suggests that some *P. alliivorans* strains persist in weed reservoirs without relying on onion-specific adaptations (e.g., *alt*) while retaining the ability to infect onion.

Our genomic analysis also supports this hypothesis, as some of the weed strains were highly similar to some onion-pathogenic strains, which were isolated several years later. These results reinforce the hypothesis that weeds serve as local inoculum sources for onion disease outbreaks. This aligns with previous research on onion, where *P. viridiflava* (the pathogen causing bacterial streak and bulb rot of onion) was shown to cause severe disease, with a high percentage of weed-isolated strains being pathogenic (49). Collectively, these observations highlight the importance of effective weed management to prevent the spread of pathogenic *P. alliivorans* strains.

Complementing this generalist trait, we also observed host-associated genetic differentiation. For example, the *alt* gene cluster, which confers thiosulfinate tolerance (a key adaptation to onion’s antimicrobial defenses), is enriched in onion-derived strains (92% of *alt*-positive strains are onion isolates). This suggests that onion-derived strains may have undergone selection to retain the *alt* cluster, whereas weed-associated strains, which are not exposed to onion’s chemical defenses, are less likely to carry it. Together, these data indicate that *P. alliivorans* includes both generalist strains capable of infecting multiple hosts (onion, weeds, pepper, *Brassica*) and strains with host-specific genetic signatures (e.g., *alt* cluster in onion isolates). Future cross-inoculation experiments across a broader range of host plants will help clarify the extent of host specialization and whether additional genetic determinants (e.g., effector repertoires) drive host specificity.

The study identified specific virulence factors in *P. alliivorans* strains, with the Hrp1-T3SS standing out as a key factor in causing disease on red onion scales. We found the Hrp1-T3SS to be essential for inducing symptoms on red onion scales, underscoring its importance in the pathogenicity of *P. alliivorans*. This finding aligns with the well-established role of Hrp1-T3SS in the *P. syringae* species complex, where it functions as a conserved pathogenicity cluster: the *hrp/hrc* gene cluster encodes a nanomachine that translocates effector proteins into host cells to suppress plant immunity and promote infection (41). Like the effectors of *P. syringae* that modulate plant immunity (50), *P. alliivorans* relies on conserved T3Es such as *avrE* to suppress plant defenses, highlighting the evolutionary conservation of effector-mediated pathogenicity in pseudomonads. In contrast, rhizobium-T3SS, T2SSs, and the *alt* cluster did not significantly affect *P. alliivorans*’ ability to cause symptoms on red onion scales, indicating potential redundancy or alternative mechanisms of virulence.

The *alt* clusters in various onion-adapted bacterial pathogens, such as *P. ananatis* and *B. gladioli* pv. *alliicola*, play a role in allicin tolerance (46, 47). This study showed that the allicin tolerance function is also present in *P. alliivorans*. The evolutionary dynamics of the *alt* cluster, however, reveal deeper parallels with the multicomponent allicin resistance mechanisms described in *P. fluorescens* PfAR-1, a garlic-associated strain with three genomic islands (GI1, GI2, and GI3) containing repeat regions (RE1, RE2, and RE3) acquired via HGT (51), as well as the *alt* clusters in *Burkholderia* onion pathogens (e.g., *B. gladioli*, *B. cepacia*, and *B. orbicola*) that contribute to allicin tolerance and growth in onion extracts (47) and the *alt* cluster in *P. ananatis* essential for colonizing necrotized onion tissue (46). These regions in PfAR-1, like the *alt* clusters in *P. alliivorans*, are enriched in redox-related genes that collectively counteract the thiol-oxidizing effects of allicin, supporting a conserved multi-gene defense strategy (51).

Phylogenetic analysis of *P. alliivorans alt* clusters highlights independent HGT events: Pa95_1 and Pa91_321 clusters align with those from *P. fluorescens* PfAR1-1/PfAR1-2 and *P. syringae* pv. *tomato* DC3000, respectively (Fig. 3), mirroring the diverse donor origins of PfAR-1′s repeat regions (REs) (51). This divergence reinforces that *alt* clusters are not inherited from a common *P. alliivorans* ancestor but are repeatedly acquired from distinct sources, a pattern also observed in PfAR-1, where REs show homology to genes from multiple plant-associated pseudomonads (51). The presence of two distinct *alt* clusters in Pa99_1 further supports that retaining multiple copies may provide an advantage, perhaps boosting thiosulfinate tolerance or supporting colonization of diverse niches with varying antimicrobial pressures, analogous to PfAR-1′s retention of three REs to reinforce resistance in garlic’s hostile chemical environment (51). The HGT is strongly supported by the *alt* cluster’s lower GC content (54.21% vs. 59.15% in the core genome of *P. alliivorans*) and association with IS66 transposases, traits shared with PfAR-1′s REs (low GC, flanked by mobile genetic elements) (51).

Functionally, the role of *alt* cluster in *P. alliivorans* aligns with the REs in PfAR-1, as both confer *in vitro* tolerance to allicin (51). Homologs of these genes in *P. alliivorans alt* clusters indicate a conserved mechanism, collectively enabling survival in onion tissues rich in thiosulfinates. The uneven distribution of *alt* clusters (92% in onion isolates) reflects host-specific selection. Weed isolates, lacking exposure to onion’s thiosulfinates, lose the cluster over time, highlighting its role in niche adaptation. This plasticity, gaining *alt* clusters via HGT in onion niches and losing them in non-onion hosts, underscores the cluster’s significance in the pathogen’s ability to colonize chemically defended hosts.

Overall, this study provides a foundation for targeted future research to improve crop health and promote sustainable agricultural methods, including: (i) developing diagnostic tools targeting the Hrp1-T3SS for the rapid detection of pathogenic *P. alliivorans* strains, (ii) designing inhibitors of the Hrp1-T3SS to disrupt virulence, (iii) integrating weed management strategies to reduce reservoirs of pathogenic strains, as our data confirm weeds as potential sources of onion infection, and (iv) exploring host resistance breeding against Hrp1-T3SS-dependent pathogenicity mechanisms.

## Data Availability

Final assemblies were deposited in the NCBI database under BioProject PRJNA1069880.

## AFTER EPUB

*[This article was published on 25 November 2025 with typographical errors in the text and tables. The errors were corrected in the current version, posted on 1 December 2025.]*

## SUPPLEMENTAL MATERIAL

Supplemental material aem.01643-25-s0001.docx Tables S1 to S3; Fig. S1 and S2.

ASM does not own the copyrights to Supplemental Material that may be linked to, or accessed through, an article. The authors have granted ASM a non-exclusive, world-wide license to publish the Supplemental Material files. Please contact the corresponding author directly for reuse.
