# Supplementary material for: Characterization of Pseudomonas alliivorans strains isolated from Georgia, USA: insights into genomic diversity and pathogenicity in onions
Source: Appl Environ Microbiol. 2025 Nov 25;91(12):e01643-25. doi: 10.1128/aem.01643-25 (PMC12724166; doi:10.1128/aem.01643-25)
Supplement: Supplemental material — Tables S1 to S3; Fig. S1 and S2. [file aem.01643-25-s0001.docx]

***Supplementary Materials***

**Characterization of *Pseudomonas* *alliivorans* Strains Isolated from Georgia, USA: Insights into Genomic Diversity and Pathogenicity in Onions**

Mei Zhao, ^a,b*^ Michelle Pena MacLellan, ^b*^ Anuj Lamichhane, ^b^ Sujan Paudel, ^c^ Ron Gitaitis, ^b^ Brian Kvitko, ^c^ and Bhabesh Dutta^b#^

^a^Department of Plant Pathology, College of Plant Protection, China Agricultural University, Beijing, P. R. China

^b^Department of Plant Pathology, University of Georgia, Tifton, GA, USA

^c^Department of Plant Pathology, University of Georgia, Athens, GA, USA

#Address correspondence to Bhabesh Dutta, bhabesh@uga.edu

*These authors contributed equally to the manuscript

### Table S1 Plasmid-related contigs predicted in *Pseudomonas alliivorans* strains.

| Strain | Number of plasmid-related contigs | Contig name | | | | | | |
| --- | --- | --- | --- | --- | --- | --- | --- | --- |
| 20GA0068 | 4 | Pa20_1_NODE_19_length_8393_cov_79.841440  Pa20_1_NODE_21_length_2299_cov_102.866787  Pa20_1_NODE_22_length_1639_cov_111.682458  Pa20_1_NODE_25_length_1231_cov_271.154246 | | | | | | |
| 20GA0069 | 4 | Pa20_2_NODE_16_length_8393_cov_83.388548  Pa20_2_NODE_18_length_2299_cov_107.391089  Pa20_2_NODE_19_length_1639_cov_112.419334  Pa20_2_NODE_22_length_1231_cov_285.772097 | | | | | | |
| 20GA0070 | 4 | Pa20_3_NODE_17_length_8393_cov_69.428099  Pa20_3_NODE_19_length_2299_cov_89.462196  Pa20_3_NODE_20_length_1639_cov_94.615877  Pa20_3_NODE_23_length_1231_cov_250.237435 | | | | | | |
| 20GA0080 | 3 | Pa20_5_NODE_18_length_5904_cov_190.046336  Pa20_5_NODE_20_length_2052_cov_529.330127  Pa20_5_NODE_22_length_1235_cov_479.136442 | | | | | | |
| 20GA0081 | 3 | Pa20_6_NODE_11_length_8276_cov_81.093020  Pa20_6_NODE_12_length_7004_cov_86.532265  Pa20_6_NODE_14_length_1466_cov_108.125990 | | | | | | |
| 20GA0082 | 6 | Pa20_7_NODE_11_length_8276_cov_75.277266  Pa20_7_NODE_12_length_7013_cov_94.516148  Pa20_7_NODE_13_length_2743_cov_67.190923  Pa20_7_NODE_14_length_2320_cov_96.172537  Pa20_7_NODE_15_length_1466_cov_98.380130  Pa20_7_NODE_16_length_1453_cov_137.092297 | | | | | | |
| 20GA0083 | 6 | Pa20_8_NODE_22_length_8513_cov_149.108345  Pa20_8_NODE_23_length_8343_cov_153.807126  Pa20_8_NODE_27_length_1540_cov_282.989064  Pa20_8_NODE_30_length_1150_cov_150.375582  Pa20_8_NODE_31_length_1124_cov_641.752627  Pa20_8_NODE_32_length_1094_cov_251.905605 | | | | | | |
| 20GA0084 | 6 | Pa20_9_NODE_17_length_12537_cov_75.348977  Pa20_9_NODE_18_length_8729_cov_60.613153  Pa20_9_NODE_19_length_8343_cov_68.299881  Pa20_9_NODE_21_length_4105_cov_116.997766  Pa20_9_NODE_24_length_1173_cov_128.937956  Pa20_9_NODE_26_length_1073_cov_127.595382 | | | | | | |
| 20GA0148 | 6 | Pa20_10_NODE_26_length_8608_cov_68.537921  Pa20_10_NODE_27_length_8342_cov_79.735123  Pa20_10_NODE_28_length_4739_cov_105.016517  Pa20_10_NODE_29_length_2052_cov_258.332658  Pa20_10_NODE_30_length_1769_cov_146.822104  Pa20_10_NODE_31_length_1732_cov_96.676133 | | | | | | |
| 20GA0149 | 6 | Pa20_11_NODE_25_length_8608_cov_55.256125  Pa20_11_NODE_26_length_8342_cov_70.106188  Pa20_11_NODE_27_length_4739_cov_95.086015  Pa20_11_NODE_28_length_2052_cov_226.307342  Pa20_11_NODE_29_length_1769_cov_123.195626  Pa20_11_NODE_30_length_1732_cov_95.030211 | | | | | | |
| 20GA0198 | 2 | Pa20_12_NODE_14_length_8306_cov_79.632470  Pa20_12_NODE_17_length_1307_cov_116.418699 |  |  |  |  |  |  |
| 20GA0201 | 6 | Pa20_13_NODE_18_length_8393_cov_78.543447  Pa20_13_NODE_20_length_4691_cov_96.141526  Pa20_13_NODE_23_length_2299_cov_92.877588  Pa20_13_NODE_24_length_1639_cov_107.658131  Pa20_13_NODE_25_length_1235_cov_265.803972  Pa20_13_NODE_26_length_1231_cov_275.434142 | | | | | | |
| 20GA0207 | 7 | Pa20_14_NODE_20_length_8488_cov_67.688741  Pa20_14_NODE_21_length_8274_cov_75.053407  Pa20_14_NODE_23_length_2265_cov_69.102377  Pa20_14_NODE_24_length_1752_cov_89.549851  Pa20_14_NODE_25_length_1635_cov_136.547497  Pa20_14_NODE_26_length_1613_cov_100.720052  Pa20_14_NODE_28_length_1186_cov_142.752029 | | | | | | |
| 20GA0227 | 8 | Pa20_15_NODE_14_length_10552_cov_90.905776  Pa20_15_NODE_15_length_9188_cov_92.476896  Pa20_15_NODE_16_length_8276_cov_78.060936  Pa20_15_NODE_17_length_6950_cov_88.097337  Pa20_15_NODE_18_length_3240_cov_81.095479  Pa20_15_NODE_19_length_2463_cov_93.068734  Pa20_15_NODE_22_length_1466_cov_101.871850  Pa20_15_NODE_23_length_1090_cov_69.717670 | | | | | | |
| 20GA0228 | 7 | Pa20_16_NODE_16_length_8124_cov_85.898305  Pa20_16_NODE_17_length_7264_cov_95.815500  Pa20_16_NODE_18_length_3046_cov_88.976086  Pa20_16_NODE_19_length_3039_cov_99.021607  Pa20_16_NODE_20_length_2956_cov_94.572074  Pa20_16_NODE_21_length_2163_cov_85.357143  Pa20_16_NODE_22_length_1219_cov_163.683012 | | | | | | |
| 20GA0233 | 3 | Pa20_17_NODE_11_length_8247_cov_86.556463  Pa20_17_NODE_13_length_1781_cov_166.134390  Pa20_17_NODE_14_length_1052_cov_150.299487 | |  |  |  |  |  |
| 20GA0235 | 9 | Pa20_18_NODE_23_length_4519_cov_89.837911  Pa20_18_NODE_25_length_4306_cov_118.453299  Pa20_18_NODE_26_length_2674_cov_156.718521  Pa20_18_NODE_28_length_1795_cov_93.541327  Pa20_18_NODE_29_length_1479_cov_182.402996  Pa20_18_NODE_30_length_1378_cov_106.029977  Pa20_18_NODE_31_length_1338_cov_86.077716  Pa20_18_NODE_33_length_1081_cov_95.426295  Pa20_18_NODE_34_length_1061_cov_146.454268 | | | | | | |
| 20GA0237 | 3 | Pa20_19_NODE_18_length_8343_cov_149.656057  Pa20_19_NODE_19_length_3536_cov_160.805724  Pa20_19_NODE_21_length_1867_cov_153.753073 | | | | | | |
| 21GA0411 | 4 | GA0411_NODE_13_length_82124_cov_105.439931  GA0411_NODE_18_length_9308_cov_1538.745856  GA0411_NODE_19_length_8168_cov_92.998423  GA0411_NODE_21_length_1245_cov_182.118151 | | | | | | |
| 21GA0412 | 7 | GA0412_NODE_17_length_64877_cov_93.623117  GA0412_NODE_19_length_8712_cov_120.337001  GA0412_NODE_20_length_8340_cov_117.746703  GA0412_NODE_22_length_2415_cov_117.685629  GA0412_NODE_23_length_2129_cov_225.760721  GA0412_NODE_25_length_1411_cov_132.040480  GA0412_NODE_26_length_1084_cov_267.383317 | | | | | | |
| 21GA0416 | 7 | GA0416_NODE_18_length_64877_cov_96.880957  GA0416_NODE_21_length_8712_cov_96.941170  GA0416_NODE_22_length_8340_cov_96.568255  GA0416_NODE_24_length_2415_cov_98.346022  GA0416_NODE_25_length_2129_cov_194.013645  GA0416_NODE_27_length_1411_cov_105.835082  GA0416_NODE_28_length_1084_cov_224.369414 | | | | | | |
| 21GA0420 | 7 | GA0420_NODE_13_length_8166_cov_96.064782  GA0420_NODE_14_length_7081_cov_108.746145  GA0420_NODE_17_length_6102_cov_107.014772  GA0420_NODE_18_length_3094_cov_142.779914  GA0420_NODE_19_length_2635_cov_92.771697  GA0420_NODE_22_length_1312_cov_87.816194  GA0420_NODE_24_length_1044_cov_93.112720 | | | | | | |
| 21GA0421 | 4 | GA0421_NODE_18_length_8340_cov_96.522633  GA0421_NODE_20_length_4721_cov_105.976529  GA0421_NODE_22_length_1570_cov_207.282652  GA0421_NODE_23_length_1549_cov_333.845788 | | | | | | |
| 21GA0425 | 3 | GA0425_NODE_19_length_8390_cov_91.958309  GA0425_NODE_21_length_2163_cov_104.302972  GA0425_NODE_28_length_1024_cov_178.972545 | | | | | | |
| 21GA0426 | 6 | GA0426_NODE_19_length_12537_cov_66.796972  GA0426_NODE_20_length_8343_cov_65.591330  GA0426_NODE_22_length_2053_cov_209.795040  GA0426_NODE_23_length_1947_cov_76.708021  GA0426_NODE_26_length_1247_cov_102.566667  GA0426_NODE_27_length_1148_cov_114.820728 | | | | | | |
| 21GA0427 | 4 | GA0427_NODE_21_length_11101_cov_108.692308  GA0427_NODE_22_length_8345_cov_103.777606  GA0427_NODE_24_length_2225_cov_107.403631  GA0427_NODE_25_length_2052_cov_612.858228 | | | | | | |
| 21GA0475 | 7 | GA0475_NODE_29_length_8634_cov_92.720930  GA0475_NODE_30_length_8342_cov_97.269985  GA0475_NODE_31_length_5675_cov_106.013755  GA0475_NODE_35_length_2550_cov_96.723817  GA0475_NODE_37_length_1831_cov_105.562144  GA0475_NODE_38_length_1572_cov_124.711706  GA0475_NODE_40_length_1421_cov_471.319196 | | | | | | |
| 21GA0476 | 5 | GA0476_NODE_18_length_8855_cov_101.371497  GA0476_NODE_19_length_8245_cov_110.744893  GA0476_NODE_20_length_2691_cov_106.062357  GA0476_NODE_22_length_2118_cov_184.546301  GA0476_NODE_23_length_1611_cov_235.870926 | | | | | | |
| 21GA0477 | 0 |  | | | | | | |
| 21GA0478 | 3 | GA0478_NODE_12_length_8168_cov_106.908187  GA0478_NODE_13_length_4385_cov_122.901114  GA0478_NODE_15_length_2063_cov_199.880161 | | | | | | |
| 21GA0479 | 7 | GA0479_NODE_19_length_8246_cov_97.396492  GA0479_NODE_20_length_5712_cov_102.163265  GA0479_NODE_22_length_2393_cov_96.731434  GA0479_NODE_23_length_2053_cov_450.195850  GA0479_NODE_25_length_1652_cov_181.895238  GA0479_NODE_26_length_1614_cov_198.940794  GA0479_NODE_27_length_1613_cov_95.766276 | | | | | | |
| 21GA0480 | 8 | GA0480_NODE_27_length_8391_cov_96.103448  GA0480_NODE_28_length_7567_cov_100.259279  GA0480_NODE_29_length_3032_cov_298.581049  GA0480_NODE_30_length_1842_cov_110.024363  GA0480_NODE_31_length_1613_cov_106.625651  GA0480_NODE_36_length_1396_cov_108.205459  GA0480_NODE_37_length_1340_cov_81.566904  GA0480_NODE_39_length_1188_cov_165.884788 | | | | | | |
| 21GA0481 | 4 | GA0481_NODE_23_length_8336_cov_105.589326  GA0481_NODE_24_length_2535_cov_100.157038  GA0481_NODE_25_length_2054_cov_181.651492  GA0481_NODE_26_length_1861_cov_466.799888 | | | | | | |
| 21GA0482 | 4 | GA0482_NODE_22_length_8336_cov_88.263758  GA0482_NODE_23_length_2535_cov_84.434906  GA0482_NODE_24_length_2054_cov_148.952959  GA0482_NODE_25_length_1861_cov_368.834081 | | | | | | |
| 21GA0483 | 4 | GA0483_NODE_20_length_8336_cov_110.747890  GA0483_NODE_21_length_2535_cov_99.813670  GA0483_NODE_22_length_2054_cov_189.045524  GA0483_NODE_23_length_1861_cov_492.833520 | | | | | | |
| 21GA0484 | 4 | GA0484_NODE_23_length_8336_cov_102.654463  GA0484_NODE_24_length_2535_cov_98.660293  GA0484_NODE_25_length_2054_cov_171.978756  GA0484_NODE_26_length_1861_cov_457.653587 | | | | | | |
| 21GA0485 | 6 | GA0485_NODE_26_length_8608_cov_89.722659  GA0485_NODE_27_length_8342_cov_95.267728  GA0485_NODE_28_length_4742_cov_108.402144  GA0485_NODE_31_length_2052_cov_307.229873  GA0485_NODE_32_length_1769_cov_182.511229  GA0485_NODE_33_length_1732_cov_111.444109 | | | | | | |
| 21GA0486 | 4 | GA0486_NODE_22_length_8336_cov_102.404374  GA0486_NODE_23_length_2535_cov_102.699349  GA0486_NODE_24_length_2054_cov_186.101163  GA0486_NODE_25_length_1861_cov_458.927130 | | | | | | |
| 21GA0487 | 4 | GA0487_NODE_23_length_8336_cov_105.872103  GA0487_NODE_24_length_2535_cov_100.333605  GA0487_NODE_25_length_2054_cov_168.710167  GA0487_NODE_26_length_1861_cov_440.084081 | | | | | | |
| 21GA0488 | 4 | GA0488_NODE_22_length_8336_cov_106.465351  GA0488_NODE_23_length_2535_cov_109.761188  GA0488_NODE_24_length_2054_cov_195.115326  GA0488_NODE_25_length_1861_cov_481.807735 | | | | | | |
| 21GA0490 | 6 | GA0490_NODE_29_length_8343_cov_83.980048  GA0490_NODE_32_length_3540_cov_192.537973  GA0490_NODE_33_length_3049_cov_179.782974  GA0490_NODE_34_length_1863_cov_65.455207  GA0490_NODE_35_length_1847_cov_149.242373  GA0490_NODE_36_length_1726_cov_85.206792 | | | | | | |
| 21GA0531 | 8 | GA0531_NODE_32_length_64808_cov_74.260061  GA0531_NODE_49_length_8712_cov_110.921019  GA0531_NODE_50_length_8340_cov_78.022098  GA0531_NODE_52_length_7157_cov_98.206921  GA0531_NODE_57_length_2415_cov_98.106929  GA0531_NODE_58_length_2129_cov_196.675926  GA0531_NODE_60_length_1411_cov_38.282609  GA0531_NODE_62_length_1084_cov_259.086395 | | | | | | |
| 21GA0534 | 7 | GA0534_NODE_17_length_64877_cov_95.133025  GA0534_NODE_19_length_8712_cov_88.751013  GA0534_NODE_20_length_8340_cov_95.773078  GA0534_NODE_22_length_2415_cov_91.410607  GA0534_NODE_23_length_2129_cov_171.759259  GA0534_NODE_25_length_1411_cov_101.410045  GA0534_NODE_27_length_1084_cov_198.393247 | | | | | | |
| 21GA0535 | 7 | GA0535_NODE_18_length_64877_cov_113.475787  GA0535_NODE_21_length_8712_cov_97.560741  GA0535_NODE_22_length_8340_cov_100.028038  GA0535_NODE_24_length_2415_cov_93.511976  GA0535_NODE_25_length_2129_cov_190.840156  GA0535_NODE_27_length_1411_cov_114.266867  GA0535_NODE_29_length_1084_cov_216.298908 | | | | | | |
| 21GA0536 | 7 | GA0536_NODE_17_length_64877_cov_95.152546  GA0536_NODE_19_length_8712_cov_95.559467  GA0536_NODE_20_length_8340_cov_98.269455  GA0536_NODE_22_length_2415_cov_95.832763  GA0536_NODE_23_length_2129_cov_176.418616  GA0536_NODE_25_length_1411_cov_112.149925  GA0536_NODE_26_length_1084_cov_208.080437 | | | | | | |
| 21GA0537 | 7 | GA0537_NODE_21_length_64877_cov_97.872145  GA0537_NODE_25_length_8712_cov_126.615055  GA0537_NODE_26_length_8340_cov_109.749198  GA0537_NODE_28_length_2415_cov_117.145851  GA0537_NODE_29_length_2129_cov_241.510721  GA0537_NODE_31_length_1411_cov_89.800600  GA0537_NODE_33_length_1084_cov_280.341609 | | | | | | |
| 21GA0553 | 7 | GA0553_NODE_31_length_8608_cov_85.066346  GA0553_NODE_32_length_8342_cov_74.431286  GA0553_NODE_33_length_4742_cov_56.291533  GA0553_NODE_36_length_2052_cov_248.398481  GA0553_NODE_37_length_1769_cov_159.985225  GA0553_NODE_38_length_1732_cov_65.879758  GA0553_NODE_41_length_1206_cov_118.193091 | | | | | | |
| 21GA0555 | 13 | GA0555_NODE_51_length_8608_cov_99.269605  GA0555_NODE_52_length_8342_cov_64.888823  GA0555_NODE_54_length_7115_cov_85.511935  GA0555_NODE_57_length_4437_cov_25.304587  GA0555_NODE_63_length_2429_cov_35.448554  GA0555_NODE_64_length_2079_cov_22.930070  GA0555_NODE_65_length_2052_cov_234.761519  GA0555_NODE_66_length_1874_cov_28.672231  GA0555_NODE_69_length_1769_cov_207.840426  GA0555_NODE_70_length_1732_cov_47.181873  GA0555_NODE_72_length_1378_cov_51.461184  GA0555_NODE_74_length_1206_cov_128.133747  GA0555_NODE_75_length_1100_cov_53.062561 | | | | | | |
| 21GA0556 | 7 | GA0556_NODE_36_length_8608_cov_82.204900  GA0556_NODE_37_length_8342_cov_70.365008  GA0556_NODE_38_length_4742_cov_48.561415  GA0556_NODE_41_length_2052_cov_229.187848  GA0556_NODE_42_length_1769_cov_156.317967  GA0556_NODE_43_length_1732_cov_66.239275  GA0556_NODE_46_length_1206_cov_112.420726 | | | | | | |
| 21GA0557 | 7 | GA0557_NODE_30_length_8608_cov_97.265268  GA0557_NODE_31_length_8342_cov_86.476897  GA0557_NODE_32_length_4742_cov_65.271168  GA0557_NODE_36_length_2052_cov_296.514937  GA0557_NODE_37_length_1769_cov_197.004137  GA0557_NODE_38_length_1732_cov_81.879154  GA0557_NODE_41_length_1206_cov_135.363153 | | | | | | |
| 21GA0559 | 6 | GA0559_NODE_25_length_8608_cov_89.446958  GA0559_NODE_26_length_8342_cov_93.686661  GA0559_NODE_27_length_4742_cov_109.567631  GA0559_NODE_30_length_2052_cov_305.463797  GA0559_NODE_31_length_1769_cov_185.377069  GA0559_NODE_32_length_1732_cov_112.209063 | | | | | | |
| 21GA0560 | 6 | GA0560_NODE_27_length_8608_cov_101.797327  GA0560_NODE_28_length_8342_cov_98.003920  GA0560_NODE_29_length_4742_cov_94.520472  GA0560_NODE_32_length_2052_cov_319.209620  GA0560_NODE_33_length_1769_cov_204.042553  GA0560_NODE_34_length_1732_cov_101.683384 | | | | | | |
| 21GA0563 | 3 | GA0563_NODE_18_length_8274_cov_93.612382  GA0563_NODE_20_length_2054_cov_226.368235  GA0563_NODE_24_length_1333_cov_76.554140 | | | | | | |
| 21GA0565 | 7 | GA0565_NODE_28_length_8608_cov_91.286250  GA0565_NODE_29_length_8342_cov_86.030645  GA0565_NODE_30_length_4742_cov_84.057020  GA0565_NODE_34_length_2052_cov_288.453165  GA0565_NODE_35_length_1769_cov_182.709811  GA0565_NODE_36_length_1732_cov_93.017523  GA0565_NODE_39_length_1206_cov_123.563330 | | | | | | |
| 21GA0567 | 3 | GA0567_NODE_25_length_8247_cov_99.137914  GA0567_NODE_27_length_4071_cov_77.695543  GA0567_NODE_29_length_2053_cov_458.465587 | | | | | | |
| 21GA0568 | 9 | GA0568_NODE_35_length_8608_cov_169.497245  GA0568_NODE_36_length_8342_cov_125.047868  GA0568_NODE_38_length_2429_cov_67.351190  GA0568_NODE_40_length_2052_cov_429.804051  GA0568_NODE_41_length_1769_cov_351.235816  GA0568_NODE_42_length_1732_cov_100.137160  GA0568_NODE_43_length_1655_cov_68.382763  GA0568_NODE_47_length_1206_cov_243.822852  GA0568_NODE_48_length_1100_cov_82.916911 | | | | | | |
| 21GA0569 | 6 | GA0569_NODE_46_length_8336_cov_98.828836  GA0569_NODE_47_length_3371_cov_84.458106  GA0569_NODE_48_length_3082_cov_97.045591  GA0569_NODE_49_length_2535_cov_127.408055  GA0569_NODE_50_length_2054_cov_158.866970  GA0569_NODE_51_length_1861_cov_492.049888 | | | | | | |
| Pa03_1 | 5 | Pa03_1_NODE_13_length_8342_cov_87.579285  Pa03_1_NODE_15_length_5488_cov_82.042876  Pa03_1_NODE_17_length_2573_cov_88.873397  Pa03_1_NODE_19_length_1399_cov_168.504539  Pa03_1_NODE_20_length_1077_cov_85.348000 | | | | | | |
| Pa200_1 | 3 | Pa200_1_NODE_12_length_8168_cov_73.202790  Pa200_1_NODE_13_length_2309_cov_78.140233  Pa200_1_NODE_14_length_2216_cov_142.045348 | | | | | | |
| Pa89_2 | 3 | Pa89_2_NODE_20_length_4160_cov_103.400519  Pa89_2_NODE_23_length_1572_cov_195.267559  Pa89_2_NODE_9_length_201913_cov_114.078207 | | | | | | |
| Pa90_1 | 3 | Pa90_1_NODE_17_length_8340_cov_80.191636  Pa90_1_NODE_19_length_1713_cov_144.515281  Pa90_1_NODE_20_length_1675_cov_106.784731 | | | | | | |
| Pa90_2 | 4 | Pa90_2_NODE_19_length_8450_cov_96.950041  Pa90_2_NODE_21_length_2193_cov_99.667769  Pa90_2_NODE_22_length_1613_cov_124.308594  Pa90_2_NODE_23_length_1308_cov_125.870024 | | | | | | |
| Pa90_3 | 4 | Pa90_3_NODE_18_length_8450_cov_88.439545  Pa90_3_NODE_20_length_2193_cov_86.349716  Pa90_3_NODE_21_length_1613_cov_104.412760  Pa90_3_NODE_22_length_1384_cov_113.508034 | | | | | | |
| Pa90_4 | 4 | Pa90_4_NODE_14_length_8393_cov_101.005549  Pa90_4_NODE_16_length_2223_cov_110.717148  Pa90_4_NODE_17_length_2052_cov_354.029367  Pa90_4_NODE_18_length_1910_cov_93.957447 | | | | | | |
| Pa90_5 | 7 | Pa90_5_NODE_18_length_19116_cov_85.374179  Pa90_5_NODE_20_length_8394_cov_93.728485  Pa90_5_NODE_21_length_4462_cov_125.524287  Pa90_5_NODE_22_length_2281_cov_90.301724  Pa90_5_NODE_23_length_2052_cov_313.729620  Pa90_5_NODE_24_length_2000_cov_180.319813  Pa90_5_NODE_27_length_1337_cov_111.557143 | | | | | | |
| Pa90_7 | 4 | Pa90_7_NODE_13_length_8393_cov_83.720897  Pa90_7_NODE_15_length_2223_cov_95.752563  Pa90_7_NODE_16_length_2052_cov_314.382785  Pa90_7_NODE_17_length_1910_cov_81.364975 | | | | | | |
| Pa91_303 | 5 | Pa91_303_NODE_16_length_8340_cov_77.500059  Pa91_303_NODE_17_length_5875_cov_72.694205  Pa91_303_NODE_18_length_4291_cov_89.613194  Pa91_303_NODE_19_length_4169_cov_78.402004  Pa91_303_NODE_20_length_4131_cov_78.745683 | | | | | | |
| Pa91_304 | 5 | Pa91_304_NODE_14_length_8340_cov_77.455388  Pa91_304_NODE_15_length_5875_cov_74.909624  Pa91_304_NODE_16_length_4171_cov_94.128969  Pa91_304_NODE_17_length_4131_cov_96.787864  Pa91_304_NODE_18_length_2602_cov_78.503366 | | | | | | |
| Pa91_305 | 5 | Pa91_305_NODE_14_length_8340_cov_87.601877  Pa91_305_NODE_15_length_5875_cov_81.229907  Pa91_305_NODE_16_length_4171_cov_112.542257  Pa91_305_NODE_17_length_4132_cov_113.184710  Pa91_305_NODE_18_length_2602_cov_84.614653 | | | | | | |
| Pa91_306 | 6 | Pa91_306_NODE_16_length_8340_cov_81.709992  Pa91_306_NODE_17_length_5875_cov_73.346326  Pa91_306_NODE_18_length_4170_cov_101.103591  Pa91_306_NODE_19_length_4132_cov_104.943527  Pa91_306_NODE_20_length_2602_cov_77.102178  Pa91_306_NODE_24_length_1130_cov_212.318139 | | | | | | |
| Pa91_308 | 5 | Pa91_308_NODE_17_length_8340_cov_76.879648  Pa91_308_NODE_18_length_5875_cov_74.846844  Pa91_308_NODE_19_length_4170_cov_94.696311  Pa91_308_NODE_20_length_4132_cov_94.853514  Pa91_308_NODE_21_length_2602_cov_74.834455 | | | | | | |
| Pa91_309 | 4 | Pa91_309_NODE_13_length_8340_cov_82.802186  Pa91_309_NODE_14_length_4171_cov_100.265755  Pa91_309_NODE_15_length_4131_cov_103.929699  Pa91_309_NODE_16_length_2602_cov_84.857030 | | | | | | |
| Pa91_311 | 6 | Pa91_311_NODE_14_length_8340_cov_92.957823  Pa91_311_NODE_15_length_5875_cov_89.129527  Pa91_311_NODE_16_length_4171_cov_120.917440  Pa91_311_NODE_17_length_4132_cov_123.745993  Pa91_311_NODE_18_length_2602_cov_88.881584  Pa91_311_NODE_21_length_1307_cov_126.792683 | | | | | | |
| Pa91_312 | 5 | Pa91_312_NODE_12_length_8340_cov_163.515386  Pa91_312_NODE_13_length_5875_cov_159.147465  Pa91_312_NODE_14_length_4171_cov_185.257694  Pa91_312_NODE_15_length_4131_cov_182.638875  Pa91_312_NODE_16_length_2602_cov_162.055050 | | | | | | |
| Pa91_313 | 4 | Pa91_313_NODE_14_length_8340_cov_85.118688  Pa91_313_NODE_15_length_4291_cov_94.708353  Pa91_313_NODE_16_length_4169_cov_89.761486  Pa91_313_NODE_17_length_4131_cov_89.497287 | | | | | | |
| Pa91_314 | 4 | Pa91_314_NODE_15_length_8261_cov_78.259655  Pa91_314_NODE_17_length_2060_cov_64.690872  Pa91_314_NODE_18_length_2052_cov_421.265316  Pa91_314_NODE_20_length_1060_cov_114.537131 | | | | | | |
| Pa91_315 | 4 | Pa91_315_NODE_15_length_8340_cov_86.723655  Pa91_315_NODE_16_length_4169_cov_101.264663  Pa91_315_NODE_17_length_4132_cov_105.384217  Pa91_315_NODE_18_length_2602_cov_85.732277 | | | | | | |
| Pa91_317 | 5 | Pa91_317_NODE_15_length_8340_cov_104.952358  Pa91_317_NODE_16_length_5875_cov_105.148672  Pa91_317_NODE_17_length_4171_cov_113.807035  Pa91_317_NODE_18_length_4132_cov_113.594081  Pa91_317_NODE_19_length_2602_cov_97.791683 | | | | | | |
| Pa91_318 | 5 | Pa91_318_NODE_17_length_8340_cov_143.210645  Pa91_318_NODE_18_length_5875_cov_137.772335  Pa91_318_NODE_19_length_4171_cov_160.807767  Pa91_318_NODE_20_length_4131_cov_167.994327  Pa91_318_NODE_21_length_2602_cov_145.475644 | | | | | | |
| Pa91_319 | 4 | Pa91_319_NODE_15_length_8261_cov_97.233149  Pa91_319_NODE_17_length_2060_cov_86.976299  Pa91_319_NODE_18_length_2052_cov_565.293671  Pa91_319_NODE_20_length_1060_cov_133.929807 | | | | | | |
| Pa91_321 | 4 | Pa91_321_NODE_16_length_8391_cov_141.750827  Pa91_321_NODE_19_length_1512_cov_222.639024  Pa91_321_NODE_20_length_1469_cov_179.489943  Pa91_321_NODE_22_length_1386_cov_327.653170 | | | | | | |
| Pa91_322 | 4 | Pa91_322_NODE_16_length_8340_cov_73.239872  Pa91_322_NODE_17_length_5820_cov_102.601254  Pa91_322_NODE_18_length_2602_cov_68.466139  Pa91_322_NODE_22_length_1109_cov_85.151163 | | | | | | |
| Pa91_323 | 4 | Pa91_323_NODE_16_length_8261_cov_86.775126  Pa91_323_NODE_18_length_2060_cov_77.745840  Pa91_323_NODE_19_length_2052_cov_493.753924  Pa91_323_NODE_21_length_1060_cov_124.064090 | | | | | | |
| Pa92_1 | 6 | Pa92_1_NODE_19_length_8247_cov_78.046973  Pa92_1_NODE_21_length_3718_cov_111.152431  Pa92_1_NODE_22_length_3427_cov_75.375224  Pa92_1_NODE_23_length_3251_cov_121.467549  Pa92_1_NODE_24_length_1824_cov_157.855180  Pa92_1_NODE_26_length_1761_cov_82.096793 | | | | | | |
| Pa93_10 | 9 | Pa93_10_NODE_15_length_8247_cov_79.365690  Pa93_10_NODE_17_length_4593_cov_89.465678  Pa93_10_NODE_18_length_3695_cov_99.788834  Pa93_10_NODE_19_length_3652_cov_95.373427  Pa93_10_NODE_20_length_3245_cov_103.293561  Pa93_10_NODE_21_length_2625_cov_74.136578  Pa93_10_NODE_22_length_2226_cov_94.865054  Pa93_10_NODE_23_length_1824_cov_145.697195  Pa93_10_NODE_25_length_1386_cov_116.722689 | | | | | | |
| Pa93_11 | 3 | Pa93_11_NODE_22_length_8431_cov_91.586904  Pa93_11_NODE_23_length_8274_cov_100.711532  Pa93_11_NODE_26_length_1013_cov_421.530983 | | | | | | |
| Pa93_12 | 5 | Pa93_12_NODE_24_length_8542_cov_76.944359  Pa93_12_NODE_25_length_8247_cov_79.313191  Pa93_12_NODE_26_length_7126_cov_90.374521  Pa93_12_NODE_28_length_6096_cov_97.610400  Pa93_12_NODE_29_length_2052_cov_252.882025 | | | | | | |
| Pa93_13 | 4 | Pa93_13_NODE_14_length_14733_cov_76.791485  Pa93_13_NODE_15_length_8247_cov_78.758289  Pa93_13_NODE_19_length_1236_cov_139.278689  Pa93_13_NODE_20_length_1069_cov_101.786290 | | | | | | |
| Pa93_2 | 9 | Pa93_2_NODE_25_length_11854_cov_74.916872  Pa93_2_NODE_27_length_9023_cov_75.510619  Pa93_2_NODE_28_length_8338_cov_84.375282  Pa93_2_NODE_29_length_7739_cov_107.802793  Pa93_2_NODE_31_length_6598_cov_96.278332  Pa93_2_NODE_32_length_3149_cov_81.506510  Pa93_2_NODE_36_length_1327_cov_70.948000  Pa93_2_NODE_37_length_1265_cov_179.107744  Pa93_2_NODE_38_length_1228_cov_77.055604 | | | | | | |
| Pa93_3 | 6 | Pa93_3_NODE_18_length_26998_cov_149.740760  Pa93_3_NODE_20_length_8649_cov_72.700887  Pa93_3_NODE_21_length_8247_cov_79.315233  Pa93_3_NODE_24_length_3035_cov_177.997972  Pa93_3_NODE_25_length_2520_cov_86.399509  Pa93_3_NODE_26_length_1623_cov_145.952781 | | | | | | |
| Pa93_300 | 5 | Pa93_300_NODE_21_length_8247_cov_75.060908  Pa93_300_NODE_27_length_1950_cov_180.896957  Pa93_300_NODE_28_length_1494_cov_142.536344  Pa93_300_NODE_31_length_1158_cov_148.486586  Pa93_300_NODE_33_length_1089_cov_88.297431 | | | | | | |
| Pa93_301 | 5 | Pa93_301_NODE_14_length_14733_cov_152.798649  Pa93_301_NODE_15_length_8247_cov_154.722970  Pa93_301_NODE_17_length_1688_cov_184.708256  Pa93_301_NODE_20_length_1328_cov_191.653877  Pa93_301_NODE_21_length_1236_cov_286.987058 | | | | | | |
| Pa93_302 | 4 | Pa93_302_NODE_24_length_8542_cov_74.917543  Pa93_302_NODE_25_length_7126_cov_101.479075  Pa93_302_NODE_27_length_6069_cov_95.059579  Pa93_302_NODE_28_length_2052_cov_253.453671 | | | | | | |
| Pa93_303 | 4 | Pa93_303_NODE_23_length_8542_cov_68.144832  Pa93_303_NODE_24_length_7126_cov_99.629309  Pa93_303_NODE_26_length_6069_cov_93.111649  Pa93_303_NODE_27_length_2052_cov_238.877975 | | | | | | |
| Pa93_304 | 5 | Pa93_304_NODE_20_length_8393_cov_83.635183  Pa93_304_NODE_21_length_8380_cov_87.454775  Pa93_304_NODE_23_length_2054_cov_180.897319  Pa93_304_NODE_26_length_1175_cov_180.476321  Pa93_304_NODE_27_length_1019_cov_145.893843 | | | | | | |
| Pa93_305 | 5 | Pa93_305_NODE_21_length_8393_cov_90.797993  Pa93_305_NODE_22_length_8380_cov_90.938817  Pa93_305_NODE_24_length_2054_cov_175.772382  Pa93_305_NODE_27_length_1175_cov_188.163024  Pa93_305_NODE_28_length_1019_cov_158.917197 | | | | | | |
| Pa93_5 | 4 | Pa93_5_NODE_17_length_8247_cov_150.732941  Pa93_5_NODE_22_length_1328_cov_191.573141  Pa93_5_NODE_23_length_1236_cov_287.031061  Pa93_5_NODE_24_length_1069_cov_193.158266 | | | | | | |
| Pa93_6 | 8 | Pa93_6_NODE_22_length_8247_cov_74.165906  Pa93_6_NODE_24_length_3981_cov_105.986936  Pa93_6_NODE_26_length_2056_cov_154.946438  Pa93_6_NODE_27_length_1950_cov_171.858516  Pa93_6_NODE_28_length_1494_cov_145.232181  Pa93_6_NODE_30_length_1321_cov_91.508039  Pa93_6_NODE_32_length_1158_cov_151.237743  Pa93_6_NODE_34_length_1089_cov_81.873518 | | | | | | |
| Pa93_7 | 5 | Pa93_7_NODE_20_length_8393_cov_89.922668  Pa93_7_NODE_21_length_8380_cov_93.492593  Pa93_7_NODE_23_length_2054_cov_182.564492  Pa93_7_NODE_26_length_1175_cov_184.273224  Pa93_7_NODE_27_length_1019_cov_146.784501 | | | | | | |
| Pa93_8 | 6 | Pa93_8_NODE_16_length_8342_cov_77.788692  Pa93_8_NODE_17_length_5329_cov_73.539985  Pa93_8_NODE_18_length_4324_cov_86.488816  Pa93_8_NODE_20_length_2669_cov_68.993441  Pa93_8_NODE_21_length_1546_cov_130.382573  Pa93_8_NODE_23_length_1220_cov_228.531934 | | | | | | |
| Pa93_9 | 6 | Pa93_9_NODE_18_length_14733_cov_71.767194  Pa93_9_NODE_19_length_8247_cov_76.455430  Pa93_9_NODE_23_length_1418_cov_109.546607  Pa93_9_NODE_25_length_1328_cov_116.680256  Pa93_9_NODE_26_length_1236_cov_137.967213  Pa93_9_NODE_27_length_1069_cov_95.642137 | | | | | | |
| Pa95_1 | 3 | Pa95_1_NODE_14_length_16844_cov_102.715572  Pa95_1_NODE_15_length_7588_cov_104.865930  Pa95_1_NODE_18_length_1052_cov_84.578462 | | | | | | |
| Pa95_3 | 5 | Pa95_3_NODE_21_length_8393_cov_70.843447  Pa95_3_NODE_22_length_8380_cov_79.771287  Pa95_3_NODE_24_length_2054_cov_165.930197  Pa95_3_NODE_27_length_1175_cov_147.611111  Pa95_3_NODE_28_length_1019_cov_136.822718 | | | | | | |
| Pa95_4 | 5 | Pa95_4_NODE_20_length_8393_cov_91.410035  Pa95_4_NODE_21_length_8380_cov_90.367939  Pa95_4_NODE_23_length_2054_cov_180.852807  Pa95_4_NODE_26_length_1175_cov_185.619308  Pa95_4_NODE_27_length_1019_cov_160.002123 | | | | | | |
| Pa95_5 | 5 | Pa95_5_NODE_20_length_8393_cov_147.781936  Pa95_5_NODE_21_length_8380_cov_162.397808  Pa95_5_NODE_23_length_2054_cov_359.206373  Pa95_5_NODE_26_length_1175_cov_325.066485  Pa95_5_NODE_27_length_1019_cov_278.911890 | | | | | | |
| Pa95_7 | 3 | Pa95_7_NODE_13_length_60911_cov_37.498455  Pa95_7_NODE_14_length_8344_cov_83.221589  Pa95_7_NODE_17_length_1124_cov_148.635148 | | | | | | |
| Pa95_9 | 4 | Pa95_9_NODE_13_length_8244_cov_71.248167  Pa95_9_NODE_15_length_2469_cov_71.968645  Pa95_9_NODE_16_length_2102_cov_70.436543  Pa95_9_NODE_20_length_1184_cov_135.253839 | | | | | | |
| Pa98_1 | 2 | Pa98_1_NODE_15_length_8342_cov_82.315239  Pa98_1_NODE_16_length_6992_cov_111.102097 | | | | | | |
| Pa98_2 | 1 | Pa98_2_NODE_18_length_8308_cov_97.107692 | | | | | | |
| Pa98_3 | 0 |  | | | | | | |
| Pa99_1 | 4 | Pa99_1_NODE_16_length_61620_cov_137.212551  Pa99_1_NODE_18_length_8343_cov_69.668290  Pa99_1_NODE_22_length_1235_cov_147.025043  Pa99_1_NODE_23_length_1124_cov_120.899713 | | | | | | |
| Pa99_5 | 4 | Pa99_5_NODE_16_length_6467_cov_130.937559  Pa99_5_NODE_17_length_5552_cov_138.907032  Pa99_5_NODE_18_length_3244_cov_96.923587  Pa99_5_NODE_22_length_1043_cov_154.501035 | | | | | | |
| Pa99_6 | 5 | Pa99_6_NODE_21_length_8883_cov_93.033046  Pa99_6_NODE_22_length_8342_cov_89.113315  Pa99_6_NODE_23_length_4737_cov_124.817167  Pa99_6_NODE_24_length_1752_cov_97.153433  Pa99_6_NODE_25_length_1622_cov_101.997411 | | | | | | |

### Table S2. Oligonucleotide primers used in this study for gene deletion.

| Primer | Sequence (5’ – 3’) | Function |
| --- | --- | --- |
| 0068gspGoutF | CGGACTGCGACACTTTCATT | Screening for putative 20GA0068Δ*gspG* |
| 0068gspGoutR | GTTGATCTACCCGGCGTTTC |  |
| 0068gspG2outF | GTTCCGACACCCTCACGTT | Screening for putative 20GA0068Δ*gspG*Δ*gspG2* |
| 0068gspG2outR | ATCCGCCTGTTGATTGGTCA |  |
| 0068altoutF | GGTAACCATTCCGCGACTTC | Screening for putative 20GA0068Δ*alt* |
| 0068altoutR | GCGGCCTTGAGTCATTTGAT |  |
| 0068hrcVoutF | TTTGCTGGAGTCATTGCTGG | Screening for putative 20GA0068Δ*hrcV* |
| 0068hrcVoutR | CTGCGGCTCGGATTCTTCT |  |
| 0068hrcV2outF | CCTGCGTTACCTGCTTGAC | Screening for putative 20GA0068Δ*hrcV2* |
| 0068hrcV2outR | GAAAGTTCTGCCCTTCGGTG |  |
| 20GA0068_hrcV_outF | TGCTGGAGTCATTGCTGGAG | Screening for putative 20GA0068 Δ*hrcV* |
| 20GA0068_hrcV_outR | CTTGAGTACGACCCGGGATG |  |
| M13_F | GTAAAACGACGGCCAGT | Confirming insertion of construct into pBS46 |
| M13_R | CAGGAAACAGCTATGAC |  |

### Table S3. Synthesized dsDNA by Twist Biosciences for making deletion constructs.

| Name | Purpose | Sequence |
| --- | --- | --- |
| 0068gspG | Deleting *gspG* in 20GA0068 | GGGGACAAGTTTGTACAAAAAAGCAGGCTTAGCCAGCGACGATCGCCATCGGCGACCATCAGGTTGCCGCCGCTGGAGCCACCATCCGGATAAAACTCCACCGCCGACCCCAGGTCGGAAGCCGTCTGCATCGTCACCTGCAAATGATCGGGCCAGTGCTGTTCACGCTTTCCCGGTGCCTTGAAGGTCAGATGACGCAGGTCGAATTCAGTACGTGCCGGCTGGCCGGTCACAATGGCCCTAACCCGCGTGGCCCGCAATGCCTCAACCATGTCACCGACCGCACGACGTTCACTGGCGGTGCTCAACCCCTGTTGCAGACCGAAACCCACGAGGCCGACAGCGATGCTCATCAACACCAGCACCACCAGCATTTCCATCAGGGTAAAACCACGGCTTGCAACAGGCGATTTCATACCGGCATTTACTCCCTAGGCATCGGGATGGTTTCCTCGTTCATGAATTCAGATATTGCTGGTCAGGCTCATCAGCGGCAGCATGATCGCCAGCATGATCACAGCTACCATGCCTGCCATGACAACCGTGAGCGCCGGGACCAGTGCGGCAAGCATACGGTCGATGCCACGCTTGGCTTCGACGTCGAAGACATCGGCCACTTTGAGCAGCATGCTGTCCAGTTCGCCGGCCTGTTCGCCGACTTCGATCATTTGCAGGGCCAGATCGGGAAGCAGCGGCTGCGCGCCAAAAGCGCTTGCCAGCGTGCCGCCTCCTTTCACCGACTCGGCAGCCTGCTCCACCTGCGCCTGCAGTGCGCGATTCGTGCAGACCTGCCGGGCAATCACCAGCGCCTGCAGCAGAGCGACACCGTTGCTCATACCCAGCTTTCTTGTACAAAGTGGTCCCC |
| 0068gspG2 | Deleting *gspG2* in 20GA0068 | GGGGACAAGTTTGTACAAAAAAGCAGGCTTAGACACGCATCAGTGTGCCTCCGGCCATGGTGATCAGAGTCTGATCGCAGCGCAGCAGCAGGTGTCCCTGATCACAATCGAGGGTCTTTACGCCAGCCTTGAGTTGTCCTTCAAGACGTAATTCTGCGAGTCGGTTTTGCGCGGCCAGCAGCGCGATTGCTCGGTCACGCAGCAGGCCATTGCTCTGTGTCATGCCGCCTGCAACACGCACCGCTGCCGACATGGCCACGGCAATGATTGCCAGCGCGACAAGCACCTCGACCAGAGTGAAACCACGTTCCGTGTGTGCCTGTCCCATGGTGCCTCCTGGCGGACTACAGGGGCGCAGAATAGCACTGGTCCAGCGTTCTGATGCGAACGCAAATCACTGAATATTTCCCTCGTCCCGTTTTATAGTTTTGTTACACTGCGGCGCTGATAAGGATTATCAAGGGAAGTGGATATGGAAGTCCCTAGGCAGCTTTAAGCGCTGCCATGAAAAACCTCTTCTTCACTGCGTCCCGACAGCGTGGCTCGGCGATCATCAGCGCGTTGCTGATCGCCGCAATCGTGGCGGTGATTGCAGCAGGCATGCTCACTCGCCAAGGCGTGCTGACTCGCAGCCTTGAAGCCGAACAATCGCGTGTGCAGAGCAGTCAGCAGTTATTGGGCGGGCTTGAGATCGGTCGCCAATTGCTCTGGGACTCGCGTCAGCAGGACGCCTCGACGCGCCTCGATCAGCCCTGGGCCAGACCAATCGTCATCGACGCGTCGAATGCCAGGCCGGGTGAGTTCGAAGGGCAATTGCAGGATCAGCAGGGCAAGTTCAATCTGCGCATACCCAGCTTTCTTGTACAAAGTGGTCCCC |
| 0068alt | Deleting *alt* in 20GA0068 | GGGGACAAGTTTGTACAAAAAAGCAGGCTTAGCCCATTTCACAGCGATGCCCGCCGCAGCTCGGTAGTAATCACCCATGAAAAGCTTTTTGCACACTGAAAGCCAGCCACGCACTTCCAATTGCTTGGTCGCATTAATGATGCTGTTGTCACCAAAAAACGCAGTGGTATGACGTGGTTGAGTTGCTAGACAACCAGTCAGATGAGCCTCCAGCTAGCTGAGCGATATTTCCCACTCGCATTCTTTTTCAAGAATCAGCACAAAACTGCCCATTCGATTCCGTTGATATTAAAAATCAGCACAGACTCGTTGAAAGCCTACCAACTGGATGGTAGCTTTATTGCAGATTTTAAATTCATGCAATCAACGCCATCCCTGTGAGAATCCCATGCCTAGGCATGTGGACGTTCCTCTTCAGATAAGCCGGACGATTAGACCGGAAGAGAAACAAGCCTACCAAGTAGTAGGTTGGCTTACAAGAGAGATGACGATACCAATAGCTTTGAAAAGCTAGGCTGCATAGAGGAATTGGGAATATTGATGACAACACAGTGAGTGTCGTGACCAACAGAGTCACTTCGAAAGCGACGCCATGAAACGATCTTTCACACCTCTATGCATCGGGTGAAATTTGTGTAATTGTACGAATTGATGGCATAAGGCTGTCACCACATGGATAAAAATCAGGGAGGTTTTATGGCTGTTTATGCACCAGGGCACGTGTTGGGGCGAGATGGAGGTTCGTTTCAAGAAGTCATACCCAGCTTTCTTGTACAAAGTGGTCCCC |
| 0068hrcV | Deleting *hrcV* in 20GA0068 | GGGGACAAGTTTGTACAAAAAAGCAGGCTTACGACAGCAACATGACCCCGCTGGCCATGAGCAAGCGGGTATTGCGCTTCTGCGGCAACGGCTTCTTCGCCCGCGACCTGACGCTGCTGGCCGAGGAAACCATCGGCCGCACGCCCACGCTGCAACCGCTGTTCTTCAACGGCTTGTACCCGCTGCTGCAGAACTTGCCGCTGACACTCTGGAAAGACCTCAAGACCCGACAGAACGGTCTGCGTCTGCTGCAGGGCCTGATGGATGAGATGGCGCGTCACGAGCGCAAGGCGCTGGGCATGGACGATGTCGGGAGAATCTCGGGATGACCCCTAGGGCCTGACGGAGCACCCATGGAAACCGACAAACTTTACGAATTGCGGGTCCTGACCGGGCTGCATCGCGGCGCAGCATTGCCCCTCAACGGCGAGCAGTGGAGCATCGGCTCGTCCAGTGGTGCAGACCTTGCGCTCTATGATCCGGGCATCAAGGACCGGCACTGCATGCTGCGACTGGTCGACGAGACCTGGTCGCTGGCCAGTAGCGAGGGGCCGGTGACCGACAGCGAAGGCCACAAGGTCGAAGCGATCAGCCAACTGGAGCCCGGCACGCCGTTCGCCGTCAACGGCGTGTGGCTGTCGGTAATCAGCGCCAACACCGAATGGCCTGCCGAGGATGAAGAAGAACCCCCTCAGGCTACCCAGCTTTCTTGTACAAAGTGGTCCCC |
| 0068hrcV2 | Deleting *hrcV2* in 20GA0068 | GGGGACAAGTTTGTACAAAAAAGCAGGCTTACTGCGCTGGATGCCGAACCGGATATCCGGGCGCTGGCTGAAGATGTGATCCGTGAGGAAATCGATATCCGCCTGTTACTGGAACAGCAACGCGCGCAGGTGCAGCCATGAGCGCGAGTCAGGATCGTGAATGCGTCGAGCTGCTCAAGGGGCTTGGCGATCTCTATCGTCGCTGCGGTCAACCGCAGCGGGCGCTGGTGATGCTGTTGATTGCCATCCAGCTGGCACCGGCCGACACCGGTCTGCTTCACAGCCTGGTGCTGGCCTTCACCAGCAGCGGTGATACCAGCAGGGCGCTGGCAGCGCTTGATCGACTGGTAAGCTATCAGGGCGAGAGCGCGGCCTTGCTGCTGTTGCGCAGTCGTGCCTTGTGGAAAGCCGGAAACAGGGACGAAGCCCGTCAGTGCTTCAGACGCTACCTGAGCGCCCGGAGGGATGAGCAATGACGTTCCCTAGGGCCTCATGACTTTTACAGTAGCTGCACTGCGTACGTTTTTCTTCAGCCTTGGCGTGCTGCTTCTGAGCAGCCATGCCAGCGCCGAGAACATCGGCAAAGGTGCCAAGGGCACGATCGATCTGGGAATCGGTGAAGGCCGGGTGCTGCATTTTTCGGCGCCCGTGGATTCGGTGATGGTCGCCGAACCGACTATTGCCGATTTGCAGGTGGTATCGCCCGGCGTGATTTACGTATTTGGCAAGGCAGCCGGGGAAACCAGTCTGATTGCTCTGGACGCTGAAGGACAGGAAACCGCGACGCTGAGCCTGTCGATCAACAGCGGCACCTCGGCCGTCTCACGGCCGCTCAAGGCGCTTCATCTACCCAGCTTTCTTGTACAAAGTGGTCCCC |
| 0068_hrcVcomp_attL | Complement *hrcV* gene | CAAATAATGATTTTATTTTGACTGATAGTGACCTGTTCGTTGCAACAAATTGATGAGCAATGCTTTTTTATAATGCCAACTTTGTACAAAAAAGCAGATGACCGGCATTATTCACCTGCTCAACCGCCTGGCACTGGCGGTCATGGGCCGCACCGAAATCGTCGGCGCAGTCATGGTCATGGCCATCGTCTTCATGATGATTCTGCCCTTGCCGACCTGGCTGGTGGACGTGCTGATCGCACTGAACATCTGTATCTCATCGCTGCTGATCGTGCTGGCGATGTACCTGCCACGTCCGCTGGCCTTCTCGACCTTTCCGGCAGTGCTGCTGCTGACCACCATGTTTCGCCTTTCGCTATCGATTGCAACGACCCGTCTGATTCTGCTGCAACAGGACGCCGGGCACATCGTCGAAGCCTTCGGCAGCTTCGTGGTGGGCGGCAACCTGGCGGTCGGTCTGGTGATCTTCCTGATTCTGACCATCGTCAACTTTCTGGTCATCACCAAAGGCTCGGAACGGGTTGCGGAAGTGGCTGCGCGCTTCACGCTGGACGCGATGCCTGGCAAACAGATGTCCATCGACAGCGACCTGCGGGCCAACCTGATCAACGTGCGCGAGGCCCGGCACCGCCGTGAACAGTTGTCCAAGGAAAGCCAGCTGTTCGGCGCCATGGACGGCGCGATGAAGTTCGTCAAGGGCGACGCCATTGCCGGTCTGGTCATCGTGTTCATCAACCTGATCGGCGGTTTCTCCATCGGCGTGCTGCAGAACGGCATGGAAGCCGGCGATGCCATGCACCTGTATTCGGTACTGACCATCGGCGACGGTCTGATTGCACAGATCCCGGCCCTGCTGATCTCGCTCACAGCGGGCATGATCATTACCCGCGTCGCACCCGACAGTCAGACGGCCGACGCCAACATCGGTCATGAAATTGCCGAGCAGCTCACCAGCCAGCCCAAGGCATGGGTCTTTTCGTCCATCGGCATGATGGGTTTCGGGCTGATTCCGGGCATGCCGACCGTGGTTTTCGCCTGCATCGCTCTGCTGTGTCTGTTCAGCGGCCTGATTCAGCTCTGGCGCATACGCCAGAAAGAGCTGCATCAGGACAGCATCGGTGCAGCCAGCAAAGGCGCGCCGGAGCTGAACGGACGTGAAGACATCCGGCGTTTCAATCCGTCACGGGCCTACCTGCTGCAATGCCATTCCAGCCATCAGGGCACGGTAGAGCTCGACAGGCTGATACATGGCATTCGTCAGCGTCGCAACACGCTGGTGCACAACTTTGGCCTGACGCTGCCCTCGTTCGACATGGAGTTCGCCGATCATTTCGAGCCCGACGAATTCCGCTTCTGCGTCTACGAAGTGCCATTCATTCGCGCCACCTTCACCAACGCGCGCGTGGCTGTCGATCATCGGGAAATCGATGAAGACGCCGGTGAGCCGGGCCGTGTCGAACGTGATGAGACACGCTGGGTCTGGGTGGACATCGATGATCCGCTGCTCAAGGAGGAAGAGCACGCCGGGGTGTCTTCGCTGGAGCTGATCCTGGAACGCATGAGCCGCGCCTTCCAGGCCACCGGACCGCAATTCATCGGTCTTCAGGAGAGCAAGGCCATCCTCAGCTGGCTGGAAAGCGAGCAGCCTGAACTGGCGCAGGAGCTGCAGCGCACCCTGCCGCTGGCGCGTTTCGCCGCCGTTCTGCAACGTCTGTCTGCCGAACTGGTGTCCTTGCGCTCGGTCAGGCCGATTGCCGAGACCCTGATCGAGCAAGGCCAGCACGAGCGCGACCTCAATGCCCTGACCGATCACGTGCGCATCGCCCTCAAGGCCCAGATCTGCCACCAGCATTGTGAAGAAAACACGCTGCATACCTGGCTGGTGACGCCGGAAACCGAAGTGCTGCTGCGTGACTCGCTGCGCCAGACCCAGAACGAATCCTACTTTGCTCTGGAGCCGGAACTGGGCAACGCGCTGGTCGAGCAACTGCGCGAAGCCTTCCCGCTGCAAGCCAGCCCCCGGCCCGTGCTGCTGGTGGCTCAAGACCTGCGCAGCCCTTTGCGCAGCCTGCTGACCGACCAGTTCCACCACGTTCCCGTGCTGTCCTTCGCCGAGCTGATCCCCACGGTTCAGGTCCAGGTGCTGGGGCGGATTGACCTGCAAGAAACCTACGACCTGATGGCCTGAACCCAGCTTTCTTGTACAAAGTTGGCATTATAAGAAAGCATTGCTTATCAATTTGTTGCAACGAACAGGTCACTATCAGTCAAAATAAAATCATTATTTG |

**
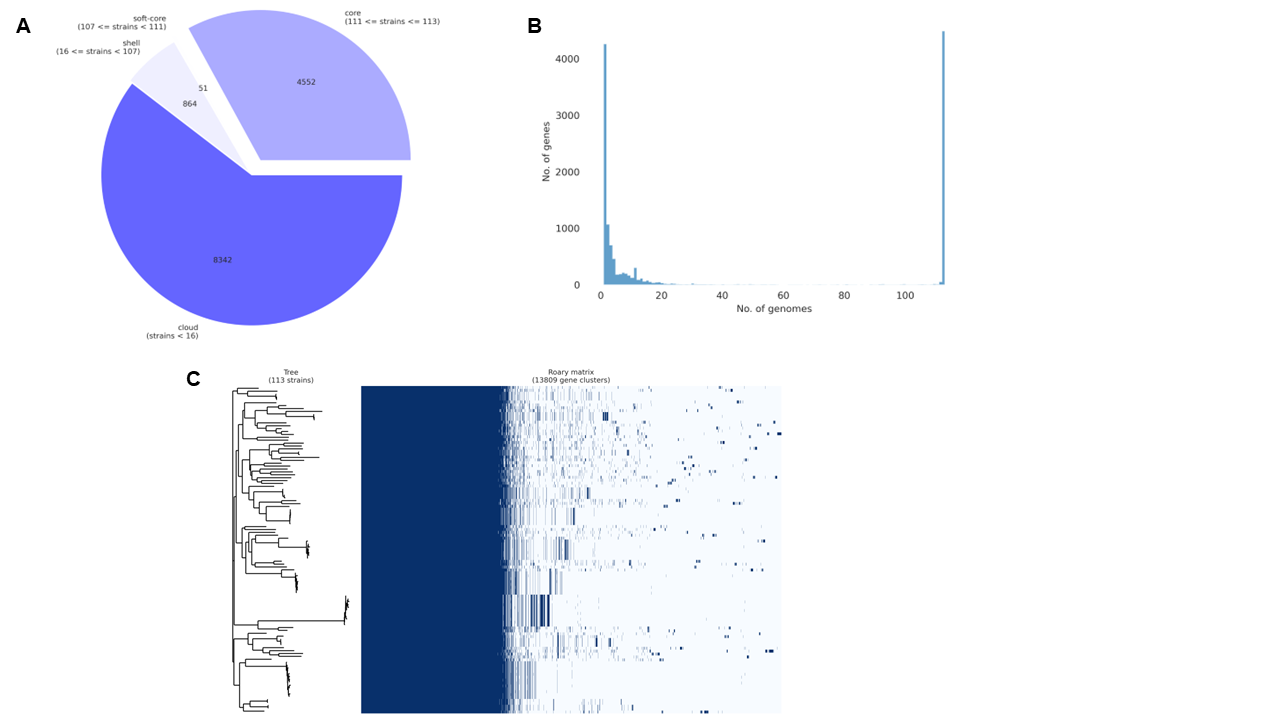
Fig S1**. Core and pan genome of 113 *Pseudomonas alliivorans* genomes isolated in Georgia, USA. (A) Pie chart representation of the pan-genome composition, showing the core genome with 4,552 genes, the soft-core genome with 51 genes, the shell genome with 864 genes, and the cloud genome with 8,342 genes, for a total of 13,809 genes. (B) Graph showing the distribution of genes in relation to the number of genomes they are present in. (C) Matrix displaying the presence and absence of core and accessory genes.

###
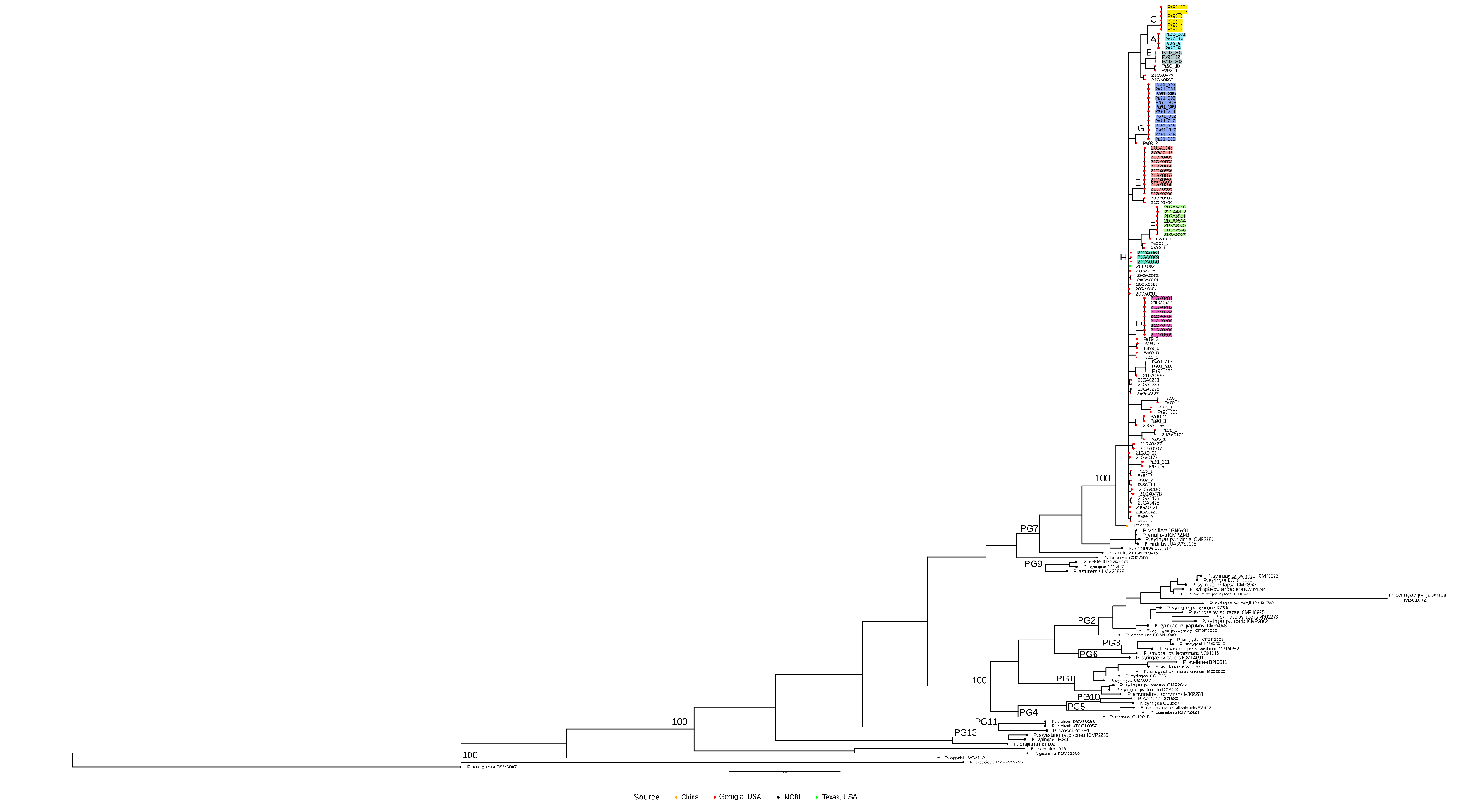
Fig S2. Maximum likelihood phylogeny based on the 1,068 single-copy orthologous protein-coding genes of 168 *Pseudomonas* strains. The phylogenetic tree was inferred using OrthoFinder v2.5.4 and drawn with R package ggtree. Tip point colors indicate strain origin: red denotes *P. alliivorans* strains isolated in Georgia (USA) from this study; green represents a *P. alliivorans* strain from Texas (USA); orange indicates a strain isolated in China; and black denotes representative, pathotype, or type strains of *Pseudomonas* species included for reference. Bootstrap values are shown in blue along the branches of the tree. PG# represent *Pseudomonas syringae* species complex phylogroups designations. Strains highlighted in A: cyan, B: gray, C: yellow, D: pink, E: orange, F: light green, G: blue, and H: dark green display short branch lengths within the subclade, indicating high genetic similarity.
